# Extraterrestrial Gynecology: Could Spaceflight Increase the Risk of Developing Cancer in Female Astronauts? An Updated Review

**DOI:** 10.3390/ijms23137465

**Published:** 2022-07-05

**Authors:** Rosa Drago-Ferrante, Riccardo Di Fiore, Fathi Karouia, Yashwanth Subbannayya, Saswati Das, Begum Aydogan Mathyk, Shehbeel Arif, Ana Paula Guevara-Cerdán, Allen Seylani, Aman Singh Galsinh, Weronika Kukulska, Joseph Borg, Sherif Suleiman, David Marshall Porterfield, Andrea Camera, Lane K. Christenson, April Elizabeth Ronca, Jonathan G. Steller, Afshin Beheshti, Jean Calleja-Agius

**Affiliations:** 1 BioDNA Laboratories, Malta Life Sciences Park, SGN 3000 San Gwann, Malta; rosa.dragoferrante@biodna.net; 2 Department of Anatomy, Faculty of Medicine and Surgery, University of Malta, MSD 2080 Msida, Malta; riccardo.difiore@um.edu.mt (R.D.F.); sherif.s.suleiman@um.edu.mt (S.S.); 3 Sbarro Institute for Cancer Research and Molecular Medicine, Center for Biotechnology, College of Science and Technology, Temple University, Philadelphia, PA 19122, USA; 4 Blue Marble Space Institute of Science, Exobiology Branch, NASA Ames Research Center, Moffett Field, CA 94035, USA; fathi.karouia@nasa.gov; 5 Space Research Within Reach, San Francisco, CA 94110, USA; 6 Center for Space Medicine, Baylor College of Medicine, Houston, TX 77030, USA; 7 Centre of Molecular Inflammation Research (CEMIR), Department of Clinical and Molecular Medicine (IKOM), Norwegian University of Science and Technology, 7491 Trondheim, Norway; yashwanth.subbannayya@ntnu.no; 8 Atal Bihari Vajpayee Institute of Medical Sciences and Dr. Ram Manohar Lohia Hospital, New Delhi 110001, India; saswati.das@cghs.nic.in; 9 Department of Obstetrics and Gynecology, HCA Healthcare/University of South Florida Morsani College of Medicine GME, Brandon Regional Hospital, Brandon, FL 33511, USA; begum.aydoganmathyk@hcahealthcare.com; 10 The Cooper Neurological Institute, Cooper University Health Care, Cherry Hill, NJ 08002, USA; sarif1@jhu.edu; 11 The G.W.C. Whiting School of Engineering, Johns Hopkins University, Baltimore, MD 21218, USA; 12 Molecular and Cellular Biology, School of Biosciences, The University of Sheffield, Western Bank, Sheffield S10 2TN, UK; apguevaracerdan1@sheffield.ac.uk; 13 School of Medicine, University of California, Riverside, CA 92521, USA; allen.seylani@medsch.ucr.edu; 14 School of Medicine, Medical Sciences and Nutrition, University of Aberdeen, Aberdeen AB24 3FX, UK; a.singh.19@abdn.ac.uk (A.S.G.); w.kukulska.19@abdn.ac.uk (W.K.); 15 Department of Applied Biomedical Science, Faculty of Health Sciences, University of Malta, MSD 2080 Msida, Malta; joseph.j.borg@um.edu.mt; 16 Department of Agricultural and Biological Engineering, Purdue University, West Lafayette, IN 47907, USA; porterf@purdue.edu; 17 Ordine dei Medici Chirurghi e degli Odontoiatri della Provincia di Brescia, 25124 Brescia, Italy; a.camera@outlook.it; 18 Department of Molecular and Integrative Physiology, University of Kansas Medical Center, 3075 HLSIC, 3901 Rainbow Blvd, Kansas City, KS 66160, USA; lchristenson@kumc.edu; 19 NASA Ames Research Center, Space Biosciences Division, Moffett Field, CA 94568, USA; april.e.ronca@nasa.gov; 20 Department of Obstetrics & Gynecology, Wake Forest School of Medicine, Winston-Salem, NC 27101, USA; 21 Department of Obstetrics & Gynecology, University of California Irvine, Orange, CA 92868, USA; jsteller@hs.uci.edu; 22 Exploration Medical Capability Element, NASA Johnson Space Center, 2400 E NASA Parkway, Houston, TX 77058, USA; 23 KBR, Space Biosciences Division, NASA Ames Research Center, Moffett Field, CA 94035, USA; 24 Stanley Center for Psychiatric Research, Broad Institute of MIT and Harvard, Cambridge, MA 02142, USA

**Keywords:** space exploration, microgravity, space radiation, astronaut health, female reproductive system, gynecological cancers

## Abstract

Outer space is an extremely hostile environment for human life, with ionizing radiation from galactic cosmic rays and microgravity posing the most significant hazards to the health of astronauts. Spaceflight has also been shown to have an impact on established cancer hallmarks, possibly increasing carcinogenic risk. Terrestrially, women have a higher incidence of radiation-induced cancers, largely driven by lung, thyroid, breast, and ovarian cancers, and therefore, historically, they have been permitted to spend significantly less time in space than men. In the present review, we focus on the effects of microgravity and radiation on the female reproductive system, particularly gynecological cancer. The aim is to provide a summary of the research that has been carried out related to the risk of gynecological cancer, highlighting what further studies are needed to pave the way for safer exploration class missions, as well as postflight screening and management of women astronauts following long-duration spaceflight.

## 1. Introduction

Human spaceflight and deep space exploration are the aspirational activities of numerous national space agencies including the European Space Agency (ESA), Japan Aerospace Exploration Agency (JAXA), National Aeronautics and Space Administration (NASA), and Canadian Space Agency (CSA), as well as many commercial and other private entities. Studies to determine the long-term physiological responses and adaptation to the space environment and further re-adaptation to the environmental conditions on Earth are of crucial importance to the safety and health of the astronauts [1].

Beyond the stressors of getting to space, such as vibration and acceleration forces, microgravity and radiation are the most significant hazards during space travel [2]. There are other sources of physiological and psychological stress during spaceflight, including circadian shifting, dietary alterations, confined spaces, and isolation [2]. The impact of spaceflight and the cosmic environment on those organ systems essential to carry out tasks in space (motor skills, cardiovascular system, and maintenance of overall bodily function) and which are essential upon return to Earth, have received the majority of focus as they relate to studies of astronaut health and wellbeing.

A long-standing concern has also been the effects of the cosmic environment on male and female reproductive health including carcinogenesis of the primary reproductive organs secondary to cosmic radiation exposure. However, this area of research lags behind. Advances in molecular biology, genetics, oncology and radiotherapy have provided more insights in recent years [2]; however, there is a paucity of research evaluating the risks of gynecological cancers, ovarian insufficiency, or infertility following spaceflight. In the present review, we focus on the effects of microgravity and radiation on the female reproductive system, particularly gynecological cancer. The aim is to provide a summary of what is known so far from studies conducted on Earth, together with the research questions and challenges requiring further study to pave the way for safer exploration class missions, as well as postflight screening and management of female astronauts following long-duration spaceflight.

## 2. Space-Environmental Factors: Microgravity and Space Radiation

Space travel, the final frontier, presents a challenge that few have faced before. Since the first flight to space in 1961, five key threats to long-duration space travel have been identified: distance from Earth, isolation and confinement, hostile/closed environments, gravity (or lack thereof), and radiation [3]. These are all areas of potentially significant concern during long-duration spaceflight. An exploration-class mission to Mars is expected to last three years, all in an enclosed environment with a small crew [4,5]. At least one of these three years would include transit time in deep space during which microgravity and radiation exposures would be elevated compared to the Martian surface. Previous literature highlights the harmful effects of such environments on several body systems, including cardiac, neurological, and immune functioning [6,7]. In addition to the elevated medical risks during exploration missions, triage and management of such conditions is complicated by limited resources, ability to evacuate, and ground communication abilities. The distance between the Earth base and Mars leads to a communication time delay of around 5–20 min one-way. This can be a particular challenge as the expertise available on Earth is no longer able to provide real-time assistance (as is the case for astronauts on the International Space Station).

### 2.1. Microgravity

Microgravity (or reduced gravity) leads to complex biological and systemic-level changes. Given that life has evolved and adapted to the presence of near-constant gravity on Earth, these changes can have consequences [3]. When exposed to altered gravity, these changes can be classified into short- or long-term effects. Over a few minutes of spaceflight, astronauts may experience space motion sickness [8]. Over a longer period, there can be remodeling of the cardiovascular and musculoskeletal systems [9]. The effects of microgravity on human physiology have been extensively investigated with the main goal of developing adequate countermeasures to minimize risks associated with long-duration spaceflight. These microgravity effects have been shown to be significant, global, and amplified with mission duration and distance from Earth. In particular, those associated with cardiovascular, musculoskeletal, neurological, and immune systems are easily diagnosed and clinically observable upon return from space.

Microgravity may synergistically combine with other factors such as radiation, additionally compromising the health and safety of the astronauts. However, though much is known about how microgravity can affect these bodily systems, research into sex/gender-related differences in the response and adaption to spaceflight as well as how microgravity can affect the female reproductive system is limited [10].

### 2.2. Space Radiation

Radiation (as waves or particles) is energy that can be classified as non-ionizing (e.g., radiowaves, microwaves, infrared) or ionizing (e.g., X- or gamma rays, protons, neutrons, heavy ions). However, ionizing radiation is the most biologically active. While popular culture imagines radiation stemming from nuclear meltdowns and atomic bombs like Chernobyl or Hiroshima, in medical practice, ionizing radiation plays critical roles in both imaging and cancer radiotherapies. In imaging, X-rays and computed tomography provide critical information allowing clinicians to tailor further interventions. In contrast, when used in radiotherapies, ionizing radiation can be used to target a tumor with a minimal impact on surrounding tissue [11]. The total radiation dose is often delivered as a series of fractionated doses over the span of 4–6 weeks. Even though the exact treatment protocol varies depending upon the type and stage of the tumor itself, typical total doses used for the treatment of gynecological cancers range from around 40–60 Gray (Gy) [12].

Ionizing radiation can impact the cells directly, where the particles impact a vital target molecule and directly transfer their energy, or indirectly, where particles impact other molecules, such as water, leading to longer lasting, very reactive free radicals. When impacting DNA, ionizing radiation can cause single-strand or double-strand DNA breaks. Double-strand DNA breaks, especially those caused by close single hits or high-energy hits, are much harder to repair. Non-rejoined breaks can lead to cell death, while incorrectly rejoined breaks can lead to mutation.

Space radiation has a complex impact on human tissues and is an etiological agent for cancer, cardiovascular diseases, central nervous system impairment, radiation sickness, and other harmful conditions [3,13]. The Earth’s magnetic field is a crucial protective element. Given the nature of ionizing space radiation, an increased rate of carcinogenesis is a primary concern for long-duration spaceflight [14]. While no increase in gynecologic cancer risk has yet been revealed in the female astronaut population [15,16], as exploration missions will be outside of low Earth orbit and for increasingly long durations, concern remains regarding the effects of even a low-dose rate accumulating over time [17]. The three main sources of ionizing space radiation are galactic cosmic radiation (GCR), solar particle events (SPE), and the Van Allen radiation belt [18]. These exposures are exceedingly different from terrestrial sources of radiation with respect to the type, energy transfer, dose rate, and total dose.

Ionizing GCR is composed of 98% nuclei and 2% electrons and positrons and constitutes a significant part of the total radiation dose [19]. The nuclear component itself is composed of hydrogen (87%; i.e., protons), helium (12%), and heavy metal nuclei (1%; including lithium, carbon, oxygen, silicon, iron, etc.) [20]. These energetically charged particles are accelerated to relativistic speeds by intra-galactic supernovae [21]. During this process, the protons and heavy-metal ions are stripped of their orbital electrons. Thus, the role of personal and spacecraft shielding will be particularly important. Electrons and positrons do not pose a major biological hazard as spacecraft shielding is sufficient to stop these particles. However, the high-energy particles (HZEs, protons, and heavy ions) are energetic enough to penetrate the shielding materials used in spacecrafts [22]. Just as these particles penetrate spacecraft shields, they also penetrate the body, raising concerns regarding the long-term health effects of GCR exposure [23].

Linear Energy Transfer (LET) is the amount of energy a particle delivers along this penetrating path to the material it travels through [24]. The value of LET determines how the particles interact with cells. The LET value is also directly proportional to how deep the particle will be able to travel. Thus, high LET radiation particles reach deeper tissues than low LET radiation particles. For example, HZEs and protons generally have high LET values as they have high energy [13,25,26]. Therefore, they are highly penetrating. This makes them very damaging to biological tissue [27,28]. It is this high-density penetrating nature that allows for these particles to induce complex double-strand breaks [13,25]. Given these differences, the International Commission on Radiological Protection created weighting factors to relate different types of radiation to cancer mortality risks (Table 1) [29]. While these weighting factors may be problematic for understanding radiation exposures in space, a conservative assumption is that a given dose of heavy ion irradiation, for example, may be at least 20 times as harmful as a given dose of x- or gamma irradiation terrestrially.

Importantly, not only do these particles penetrate spacecraft shielding and bodily tissues, but they also interact with them, leading to the generation of secondary neutron radiation and reactive oxygen species, which can be just as, if not more, biologically harmful than the primary GCR particles. All of these details lead to a complex radiation environment onboard mission spacecraft, adding to the challenge of protecting astronauts from the ionizing effects of space radiation [23,30].

Of additional concern within the interplanetary radiation environment beyond the constant exposure to GCR is the relationship between solar cycles, solar wind, and solar particle events to the overall exposure [31]. Solar particle events (SPEs) occur when particles emitted from the sun are accelerated, either close to the sun or in interplanetary space. These particles consist of mainly 95% protons, electrons, HZE ions, and alpha particles [32]. However, unlike GCR, SPE radiation is of high flux and low energy. Thus, spacecraft shielding is much more effective at blocking SPE radiation and most residual SPE radiation can be absorbed by superficial tissues. Skin doses of SPE are 5–10× higher than that of internal organs and are therefore more likely to cause skin lesions and hematological and immunological disturbances [19,23]. While SPEs can range in size, they rarely result in high total dose exposures.

Furthermore, the exposure is different between the low Earth orbit (LEO) and beyond-LEO environment during interplanetary travel, which will also be different from that on the Mars surface. Defined as 80–2000 km above Earth’s surface (and below the Van Allen Belts), the radiation environment in LEO is starkly different to that beyond LEO. The LEO is naturally shielded by Earth’s atmosphere and magnetic field. Although there can be increases in solar radiation during rare large solar particle events and coronal mass ejections (CMEs), we are largely protected from the majority of GCR [28]. In comparison to the ~1 mGy/year at sea level, estimates of around 0.3–1 mGy/day have been suggested to occur in deep space [30,33]. The projected dose received during a Mars mission (6 months of travel each way and 2 years of surface stay) could therefore result in a total cumulative dose equivalent close to 1000 mGy [17]. More recently, with radiation dosimeter readings during the cruise phase of the Mars Curiosity mission, we can expect trans Earth–Mars exposures up to 1.8 mGy/day [34]. Still, these can be subject to change based on local conditions and extreme events such as solar flares.

All of these unique features of space radiation make it very difficult to extrapolate conclusions from terrestrial radiation research for hypothesizing risk profiles in the space environment. The majority of data are derived from human studies in which inadvertent exposures to high total dose or high-dose rate, short-duration exposures to gamma irradiation that have occurred after nuclear events, or exposures in patients being treated for existing cancer with high-dose rate external beam radiation or internal gamma radiation (brachytherapy) or in non-human mammalian studies have been analyzed. However, the vast majority of animal studies use X-ray and gamma irradiation, which are equivalent to GCR exposures. Moreover, X-ray and gamma irradiation exposures predicted to be seen in spaceflight are not of clinical significance. Studies completed at Brookhaven National Laboratory, where researchers can simulate GCR by using a mix-beam of protons and heavy ions, provides a mechanism to study exposures in model mammalian systems. However, these studies are also limited to using a small number of high fractionated doses to achieve a desired total dose for exploration missions, because it would not be feasible to run daily low-dose rate exposures for long durations in order to replicate a 3-year Mars mission.

In summary, to try and predict the risk of gynecologic cancer during or following spaceflight, we must understand that with the exception of true long-duration human spaceflight studies, our current knowledge is severely limited by the characteristics of the study. These characteristics include radiation type, energy transfer, dose rate/duration of exposure, total dose, animal model, presence of atmosphere, presence of magnetosphere, personal shielding, craft/dwelling shielding, and use of antioxidants or other countermeasures.

## 3. Effect of Spaceflight on Female Reproduction

**Figure 1:**
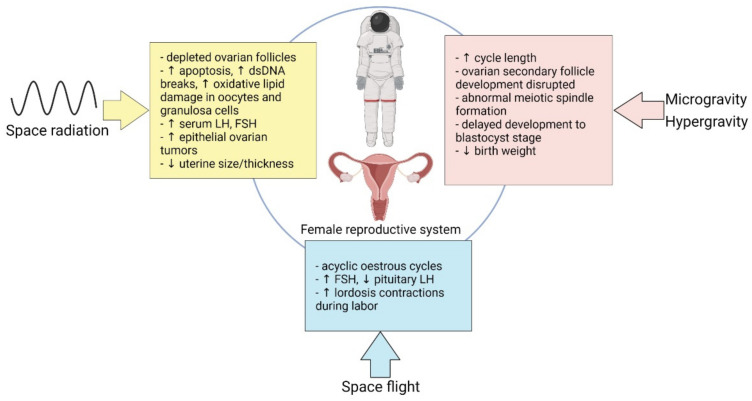
A summary of the effects of microgravity, space radiation, and space flight on the female reproductive system.

There is a paucity of evidence highlighting the effects of microgravity, space radiation, and spaceflight on the male and female reproductive systems, with significantly less for the female system based on the lack of women being exposed to GCR in the Apollo missions and past inequities. Most of our current knowledge on the female reproductive system stems from animal studies and are summarized in Figure 1. Notably, the past decade has witnessed a dramatic increase in the number of women living and working in space. NASA astronaut candidate class ratios of men to women have achieved parity since 2013, making it likely that the future of long-duration spaceflight may also feature equal numbers of men and women [16], thus warranting further study into how the cosmic environment affects reproductive health.

### 3.1. Microgravity

Microgravity exposure poses multiple female reproductive health concerns. These include effects of weightlessness on gonadal function and fertility as well secondary spaceflight stressors, such as sleep disruption, that may degrade female reproductive health during and after spaceflight. However, the scientific literature on reproductive changes in female astronauts during and after spaceflight or exposure to simulated microgravity (bedrest) remains sparse [10,16,35]. While female astronauts have successfully conceived and born children after spaceflight, detailed information on post-spaceflight fertility, pregnancy complications, and birth outcomes in women is not available. Further, female astronauts tend to delay pregnancy, making it difficult to separate the effects of spaceflight stressors from maternal aging on fertility and pregnancy outcomes. However, some animal studies attempting to understand reproductive outcomes in spaceflight have occurred, the first was in 1979 aboard an 18.5-day COSMOS 1129 mission, where a barrier between the two male and five female rats was removed on day two of orbit to allow for mating. Upon return to Earth, no pregnancies were observed. However, no pregnancies were observed in the control group of rats maintained on Earth either. It is not clear whether the absence of pregnancy was due to an inability to copulate in the weightless space environment, or to secondary more complex endocrine and/or embryonic developmental causes, or even attributable to housing/caging concerns [35,36].

Studies of female mice that were maintained on the International Space Station (ISS) for 37 days prior to euthanasia in space (eliminating re-entry stressors) revealed evidence that these female mice could be detected at different stages of the estrous cycle post-mortem, suggesting that some females were likely exhibiting estrous cyclicity [37]. Mouse embryos flown on China’s SJ-10 biosatellite completed a series of cell divisions leading to blastocoel morphology during spaceflight, but both the rate of blastocyst formation and blastocyst quality were impaired [38]. Severe DNA damage was observed in the embryonic cells, and the genome of the blastocysts developed in space was globally hypomethylated with a unique set of differentially methylated regions (DMRs). The authors suggest that these changes are similar to developmental defects, DNA damage, and epigenetic abnormalities that occur with exposure to ground-based low-dose radiation. However, given the dramatic differences already noted between cosmic radiation and that of gamma irradiation that they used in their experiments, as well as to the inability to separate out the effects of microgravity from the impact of space radiation in these studies, much remains to be determined.

While a large effort in understanding fertility outcomes is still needed, a few studies have been performed evaluating mammalian pregnancy and embryonic/prenatal development in space [39]. In 1982, COSMOS 1514 examined the effect of a 4.5-day flight exposure on gestational days 13–18 of a 21-day rat pregnancy. Dams were euthanized immediately upon landing and the fetuses exhibited neurobiological aberrations and impaired bone development. Four of five dams successfully delivered their litters postflight. Additional findings included poor maternal weight gain and evidence of fetal growth restriction in comparison to controls, possibly related to the potato diet used to provision both water and food, but no change in litter dynamics. Male and female offspring from these litters that developed to adulthood were fertile and reproduced successfully. In 1994 and 1996, two jointly sponsored NASA-National Institutes of Health missions (NIH.R1) (STS-66) and NIH.R2 (STS-70) launched pregnant (gestational day 9 and 11, respectively) dams that were returned to Earth on gestational day 20 prior to parturition. Space-flown pregnant rats gave birth at the expected time; however, they exhibited twice as many ‘lordosis’ contractions during labor coupled with decreased uterine myometrial connexin 43 (gap junction) protein expression relative to controls, suggesting changes to the uterine smooth musculature tone with exposure to microgravity. However, the duration of labor, maternal weight gain, miscarriage/stillbirth rate, litter size, neonatal birthweight, placentophagia, and maternal care patterns were not significantly different from ground controls [40,41]. Importantly, NIH.R1 and R2 offspring were flown for the second half of the rats’ gestational period, after organogenesis was complete, and returned to Earth for parturition. There have been no additional studies of mammalian pregnancy during spaceflight, and no mammal has yet given birth in space. Few studies have investigated rodent pregnancy utilizing terrestrial analogs for microgravity although past studies have exposed reproducing adult rodents to hypergravity produced via chronic acceleration [42,43,44]. This work provides strong evidence that, for some reproductive parameters such as pregnancy outcome and mammary gland metabolism, gravity load elicits a response continuum, and a single study compared responses to spaceflight and hypergravity. Analysis of mammary glands from G20 dams revealed a strong negative correlation between metabolic rate and gravity loads spanning 0, 1.5, 1.75, and 2 g. Approximately 98% of the variation in glucose oxidation and 94% of the variation in glucose incorporation into lipids was accounted for by differences in gravity ‘dose’. These data demonstrate a remarkable continuum of response across the microgravity and hypergravity environments for this reproductive parameter.

As reproductive factors such as age at first pregnancy, parity, and breastfeeding are all inversely linked with gynecologic and breast cancers terrestrially [45,46,47], more studies will be needed to explore any link between reproductive fitness after spaceflight and future risks of cancer occurring in these same organs.

### 3.2. Hormonal Modalities in Spaceflight

A unique operational consideration for premenopausal female astronauts is the use of hormonal contraception to suppress ovarian function, prevent pregnancy, and reduce menstrual flow or induce amenorrhea during pre-flight training and spaceflight. With the pathway that astronauts follow to reach candidate selection and the mission training phase, female astronaut candidates often opt to use hormonal contraception through the phases of candidate selection and training, while awaiting mission selection, during mission-specific training, and during the mission itself, potentially amounting to 11 or more years of reproductive suppression [48]. The combined oral contraceptive pill and levonorgestrel intrauterine device are the most commonly used hormonal contraceptive options.

However, it is difficult to predict how hormonal contraception use in combination with the complex deep space environmental exposure will affect female astronaut health as it relates to the intertwined nature of reproductive function on multiple organ systems. It is unknown how the environment of deep space, especially for long durations, will impact the shelf-life, pharmacokinetics, and pharmacodynamics of the contraceptive agents as well as contraceptive efficacy, menses/abnormal uterine bleeding, ovarian cysts/torsion, venous thromboembolism, cardiovascular health, musculoskeletal health during exploration-class missions, or indeed gynecological cancers.

### 3.3. Space Radiation

The likelihood of a significant acute exposure that would lead to acute radiation syndrome while a crew is in LEO is very low. However, the likelihood of an acute high-dose radiation exposure is higher for a crew traveling in interplanetary space in a minimally shielded spacecraft. As previously mentioned, SPEs of sufficient intensity to breach clinical thresholds during a trip to Mars have been recorded in the recent past. Tissue sensitivity depends on the cellular, extracellular, and stroma composition of the tissue. In general, tissues that have a large number of active stem cells are highly sensitive to radiation, whereas tissues with mainly terminally differentiated or with large amounts of supporting stroma and noncellular elements are relatively radioresistant.

In females, the ovary is extremely radiosensitive. Radiation-induced cessation of hormone production can lead to temporary or permanent infertility. The vagina is similar to other mucous membranes in terms of radiosensitivity, but the vulva, labia, and clitoris are more radiosensitive. The uterus is radioresistant. Transient sterility can occur after doses as low as 1250 mGy, although most report the threshold dose for temporary sterility as being 1700 mGy [49]. The dose required for permanent sterility in women ranges from 3500–20,000 mGy (Table 2), with lower doses needed for women older than 40 years [50,51]. Radiation damage to the female gonads is cumulative because gametogenesis essentially stops at the time of birth.

## 4. Effect of Space Travel on Cancer

Carcinogenesis is the multi-step transformation of normal cells into malignant tumors, requiring the accumulation of several genetic and epigenetic aberrations. Cancer is characterized by the continuous proliferation of tumor cells, accompanied by resistance to cell death, induction of angiogenesis, invasion, and metastasis [52].

DNA mutations altering protein-coding genes and signal transduction pathways are among the factors involved in cancer etiology. The protein-coding gene mutations involve tumor-suppressor genes, transmembrane proteins, platelet-derived growth factors, sex hormones, components of the insulin-like growth factor axis, transcription factors of the forkhead/winged helix-box transcription factor (Fox), and the SMAD families. Signal transduction pathways that are involved in carcinogenesis include sonic hedgehog (SHH), Wnt, and Notch [2]. There are also viruses like the human papillomavirus (HPV), Epstein–Barr virus, and Hepatitis B and C, which are oncogenic [53]. Recent advances in the field of tumor biology are highlighting the role of the microenvironment and the altered stress response favoring overall survival [54].

There are multiple environmental factors, such as space radiation and microgravity, which can contribute to possibly increasing any risk of tumor development and the possible underlying cellular mechanisms associated with spaceflight [55]. In addition to the studies reviewed in Moreno-Villanueva and Wu [55], more investigations involving microgravity and radiation have been carried out, as shown in Table 3 [56,57,58,59,60,61,62,63] and Table 4 [64,65,66,67,68,69,70,71,72,73], respectively. Although ionizing radiation is a known carcinogen, irradiation with particles in space differs quantitatively and qualitatively from γ-radiation or X-rays [30]. This limits our understanding of the risk of carcinogenesis, which may be possibly associated with space radiation. The biological effects of irradiation with heavy ions leading to DNA damage and repair, genomic instability, mutagenesis, chromosome aberrations, and neoplastic transformation have been documented [74]. Mouse models have generally been used for studying radiation-induced carcinogenesis, but these do not reflect the full range of complexity of cancer in humans [75]. Indeed, one main limitation for quantifying space-associated cancer risk is that there are no human data from extended exposure to space radiation, and in fact, estimation of carcinogenic risk for humans exposed to cosmic radiation is very difficult to ascertain [76,77].

**Table 3:** List of studies showing effects of microgravity on cancers.

Cancer Type	Microgravity Model	Model	Effect	Study
Breast cancer	6 min of r-µg *; PF ** maneuvers	MCF-7 cell line	Rearrangement of F-actin and tubulin, appearance of filopodia- and lamellipodia-like structures; PF-induced differential regulation of *KRT8*, *RDX*, *TIMP1*, *CXCL8* (up), *VCL*, and *CDH1* (down) genes	Nassef et al., 2019 [56]
Breast cancer	Exposure to an RPM ^##^	MCF-7 cell line	Cells formed multicellular spheroids resembling epithelial ducts; microgravity-induced differential regulation of *IL8*, *VEGFA*, *FLT1*, *ESR1* (up), *ACTB*, *TUBB*, *FN1*, *CASP9*, *CASP3*, and *PGR1* (down) genes	Kopp et al., 2016 [57]
Breast cancer	PF ** maneuvers; incubator RPM ^##^	MDA-MB-231 cells	Differential regulation of *ICAM1*, *CD44*, *ERK1*, *NFKB1*, *FAK1* (up), *ANXA2*, and *BAX* (down) genes	Nassef et al., 2019 [58]
Glioma	Exposure to an RPM ^##^	U251 cells	Induction of apoptosis; reduced FAK/RhoA/Rock and FAK/Nek2 signaling events	Deng et al., 2019 [59]
Lung cancer (non small cell)	Exposure to an RPM ^##^	NCI-H1703 (CRL-5889) cells	Formation of multicellular spheroids; spherical rearrangement of actin filaments in the outer region of cytoplasm; increased apoptosis, upregulation of *TP53*, *CDKN2A*, *RB1*, *PTEN*, and *SOX2* in stimulated adherent cells	Dietz et al., 2019 [60]
Melanoma	Exposure to a 3-D Clinostat ^#^	A375 cells	Decreased cell viability; increase in caspase 3/7 activity; reduced cell proliferation; change in cell morphology (presence of membrane blebbing lamellipodia, and stress fibers, absence of filopodia)	Przystupski et al., 2021 [61]
Thyroid cancer	Exposure to an RPM ^##^	FTC-133 cells	Cells formed multicellular spheroids; differential regulation of *ERK1*, *EGF* (up), *CTGF*, and *CAV* (down) genes in multicellular spheroids	Warnke et al., 2014 [62]
Thyroid cancer	10 day of r-µg *	FTC-133 cells	Differential expression of IL6, IL7, IL8, VEGF, TIMP1, MMP3, CCL4, and B2M (up) proteins	Riwaldt et al., 2015 [63]

* r-µg: real microgravity; ^#^ s-µg: simulated microgravity; ** PF: parabolic flight; ^##^ RPM: Random Positioning machine (simulated microgravity).

**Table 4:** List of studies showing effects of space radiation on cancer risk.

Cell Type	Radiation Model	Cell/Animal Model	Effect	Study
Lung cells	Iron ion (Fe) beam (180 MeV/nucleon; LET 300 keV/µm) for 0.1 Gy	SV40-immortalized human bronchial epithelial cells (NL20)	Progeny of Fe-irradiated cells showed elevated micronucleus formation, increased markers for DNA double-strand breaks (γ-H2AX foci), reduced cell proliferation, persistent oxidative stress, and increased colony formation.	Cao et al., 2018 [64]
Lung cells	56Fe (600 MeV/u at 0, 0.1, 0.3, 1.0 Gy) and 28Si (300 MeV/u at 0, 0.3, 1.0 Gy) high LET irradiation	Immortalized human bronchial epithelial cell line (HBEC3-KT)	Global differential CpG island methylation in response to 56Fe and 28Si ion exposure suggests a lasting impact on the epigenome relevant to lung cancer	Kennedy et al., 2018 [65]
Hematopoietic stem cells	100 cGy of 1000 MeV/n protons (LET 0.23 keV/micron); 28Si 300 MeV/n ions (LET 70 keV/micron)	Mlh1+/− mice (B6.129-Mlh1tm1Rak/NCI) representing loss of MLH1 that occurs in human hematopoietic stem cells with age	High LET 28Si ion irradiation affected hematopoietic stem cell differentiation; high LET irradiation caused early and higher incidence of tumorigenesis in Mlh1 heterozygous mice; frequent occurrence of T-cell rich B-cell (TRB) lymphomas with altered mismatch repair pathway	Patel et al., 2020 [66]
Spleen cells	0.5 Gy Proton irradiation (1-GeV; LET 0.24-keV/µm)	Murine Lewis lung carcinoma (LLC) cells-bearing C57BL/6 mice	Upregulation of genes involved in DNA repair and cell cycle, including CDK2, MCM7, CD74, and RUVBL2	Wage et al., 2015 [67]
Intestinal cells	56Fe-irradiation (1.6 Gy; energy-1000 MeV/nucleon; LET-148 keV/µm)	Intestinal tissue from Female C57BL/6J mice	56Fe-irradiation upregulated metabolites belonging to prostanoid biosynthesis and eicosanoid signaling pathways linked with cellular inflammation, which has been associated with intestinal inflammatory disease and colon cancer	Cheema et al., 2014 [68]
Liver cells	56Fe ion irradiation (1 GeV/nucleon)	CBA/CaJ mice	Higher incidence of hepatocellular carcinoma than γ-irradiated mice	Weil et al., 2009 [69]
Kidney cells	56Fe ions irradiation (1 GeV/amu, 151 keV/μm)	Aprt heterozygous (Aprt+/−) B6D2F1 mice	Increased mutant frequencies leading to DNA damage	Turker et al., 2017 [70]
Cervical cancer cells	Kept at the Russian Mir space station (40 days); American space shuttle (10 days)	HeLa cells	DNA damage	Ohnishi et al., 2002 [71]
Normal human foreskin fibroblast cells	Kept at the International Space Station (14 days)	AG1522 cells	Larger size γ-H2AX foci suggest DNA damage	Lu et al., 2017 [72]
Normal human foreskin fibroblast cells	Kept at the International Space Station (14 days)	AG1522 cells	Downregulation of miRNA Let-7a, which was found to be downregulated to γ ray and UV ray radiation in another study	Zhang et al., 2016 [73]

The standard approach for analyzing carcinogenesis is via relative biological effectiveness (RBE) studies utilizing low LET to high LET scaling factors. In addition, radiation effects ratio (RER) is a new metric that is being proposed, which compares the effects of two radiations at the same dose [78]. However, there are still important dilemmas regarding cancer risk at low-dose rates [79,80]. Therefore, in order to improve the estimation of carcinogenesis risk of long-duration space travel, there needs to be more mechanistic analysis and biological insight of radiation quality effects and long-duration exposure to low radiation dose rates. The relationship between multiple space–environmental factors that can influence the development of cancer should be investigated separately and also in combination.

To date, there have been a limited number of studies investigating the combined effect of space radiation and microgravity on cancer development. Recent findings suggest that the antiproliferative effect of simulated microgravity may lead to novel therapeutic strategies in combating cancer. In contrast, an impaired radiation-induced DNA damage response that can promote tumor initiation has been observed under microgravity conditions in vitro [55]. In studies using devices that simulate microgravity, a microgravity-associated increased radiosensitivity has been reported. However, there are currently minimal data regarding the effect of spaceflight on the capacity of the cells to repair any artificially induced DNA damage. Moreover, experiments investigating DNA damage response in simulated microgravity are not always concordant with those conducted in real microgravity [81].

Beyond the concerns of radiation-induced DNA damage, numerous molecular studies add to the hypothesis that individualized risk could be increased in the setting of microgravity, as important signaling pathways commonly involved in carcinogenesis could also be dysregulated under microgravity conditions. One example includes the phosphoinositide-3 kinase (PI3K) signaling pathway [82,83,84], which is a key driver of metabolism, cell survival, and proliferation in response to growth factor stimulation [85].

Sex-specific cancers (involving the breast, ovary, or uterus) together contribute heavily to overall cancer incidence and mortality in women. Breast cancer is the largest contributor to cancer incidence following terrestrial radiation and the three organs combined make up over 30% of the risk of exposure-induced cancer. Breast cancer is also the second largest contributor to cancer mortality following terrestrial radiation, and the three organs combined make up over 20% of the risk of exposure-induced death. Both ovarian and uterine cancers have 5-year survival rates of 49.1% and 66.3%, respectively.

Colorectal, breast, prostate, and lung cancer cells have been studied in simulated microgravity in relation to the dysregulation of the PI3K pathway. In colorectal cancer cells exposed to simulated microgravity in a Rotational Cell Culture System-High Aspect Ratio Vessel (RCCS-HARV), Akt phosphorylation was found to decrease whilst PTEN expression and activity increased, leading to the induction of apoptosis [86]. Similarly, apoptosis rate was seen to increase in lung cancer cells exposed to simulated microgravity, as tumor-suppressor genes were upregulated when cells were cultured in a random-positioning machine (RPM), and *AKT3* and *PIK3CA* expression remained unaltered.

Interestingly, a study carried out on normal and cancerous breast cells found that the paradox between weightlessness-induced apoptosis and Akt upregulation under microgravity conditions depends on whether the cells are adhered or detached. It has been concluded that survival strategies under this type of mechanical stress vary between cell types. They do in fact observe that apoptosis occurs in the cancerous cell line growing in floating organoid-like structures after 72 h due to major cytoskeletal rearrangements happening as a result of the simulated loss of gravity using an RPM [87]. These findings are supported by the study carried out on prostate cancer cells cultured in an RPM; Hybel et al. also reported that cells adhered to the culture flask significantly upregulated genes of the PI3K pathway, such as Akt and mTOR, and floating cells that formed multicellular spheroids downregulated these [88]. However, it remains unclear how accurately the different methods used to simulate microgravity reflect the results that would be observed in tissues under real microgravity, and how different equipment may affect experimental reproducibility, as conflicting results have often been reported in the past [89].

There is no definitive evidence of increased rates of carcinogenesis amongst astronauts exposed to space radiation in comparison to terrestrial controls (likely because of their overall excellent health pre-flight, low overall doses experienced to date, and very small sample sizes) [90]. However, it is reasonable to assume that space radiation would increase an astronaut’s individualized risk [30]. Furthermore, when reviewing astronaut and analog populations of aircrews exposed to cosmic radiation, Di Trolio et al. concluded that it was unclear whether increased exposure to cosmic radiation was directly linked to carcinogenic risk or whether the risk may be more attributable to lifestyle factors [91].

## 5. Gynecological Cancers and Space

### 5.1. Brief Overview on Gynecological Cancers

Gynecological cancers (GCs) arise in the female reproductive organs and include tubo-ovarian, uterine/endometrial, cervical, vaginal, and vulvar cancers [92]. GCs pose a serious global health burden due to their high incidence among women of all ages [93]. There is a high mortality rate among women with GCs, which can be attributed to several factors including lack of screening, limited awareness of specific symptoms, or even misdiagnosis.

In advanced GCs, delayed diagnosis, together with limited treatment options, are major contributing factors leading to high mortality. In the case of rare GCs (for example gestational trophoblastic neoplasia, malignant germ-cell tumors, sex cord-stromal tumors, vaginal/vulvar carcinoma, etc.), these issues are even more problematic [94]. These tumors are generally associated with an overall poor prognosis. The low incidence of each of these rare tumors, with an annual incidence of <6 per 100,000 women, poses a major hurdle in the management of patients due to limited therapy options [93].

Considerable studies have shown that the occurrence and development of GCs are related to the inactivation of tumor-suppressor genes, the activation of oncogenes, and the activation of abnormal cell signaling pathways. In addition, epigenetic processes regulate gene expression through histone modification, DNA methylation, and noncoding RNA, thereby playing a central role in the occurrence and development of GCs [95].

Ovarian cancer (OC) is an umbrella term for a heterogeneous group of tumors that are very diverse behaviorally, morphologically, and molecularly. Up to 90% of OCs are of epithelial origin [96]. OC is commonly associated with alterations in *BRCA1/2* and *TP53*, both of which are linked with a poor prognosis [97,98,99]. Typically, the onset is insidious, with no specific clinical symptoms in the early stage of the disease. To date, there is a paucity of sensitive and effective clinical screening tools for OC, with approximately 70% of cases being diagnosed at an advanced stage [100]. According to the American Cancer Society, in the United States alone, approximately 21,000 new cases of OC are diagnosed annually, accounting for 5% of all female malignancies, with a mortality rate of 62% and a five-year survival rate of 20–30% [101]. Thus, there is an urgent need for highly sensitive and specific diagnostic tools that identify OC at an earlier stage together with the development of new therapeutic approaches to improve patient survival rate.

Worldwide, cervical cancer (CC) is the second leading cause of cancer-related deaths in women [102]. Clinically, CC is associated with persistent infection with ‘high-risk’ human papillomaviruses (HPVs), particularly the subtypes HPV16 and HPV18 [103,104]. In addition, a number of other risk factors have been associated with CC, including early sexual activity [105], multiple sexual partners [106], other viral infections (such as HIV, herpes simplex virus type II), chlamydia infections [107], genetic factors (active oncogenes, including *PIK3CA*, *ATAD2*, and *CRNDE*; tumor-suppressor genes, including *TP53*, *RASSF1A*, and *NOL7*) [108], and tobacco use [109]. For the detection of pre-invasive cervical disease, Papanicolaou smears and liquid-based cytology were historically utilized as the main screening tests [110]; however, there is currently a shift in practice underway towards primary human papillomavirus (HPV) screening. Despite the continuous advances in treatment, including radiotherapy and/or chemotherapy together with surgery, early lymph node metastasis can still occur in a number of patients with CC, leading to poor prognosis. The five-year survival rate is still approximately 40% [111,112,113,114]. Although the HPV-associated carcinogenic pathway of CC is now well elucidated, further in-depth studies are necessary to aid in the discovery of novel molecular therapeutic targets that would contribute to the management of patients with advanced or recurrent CC.

Endometrial cancer (EC) is another common type of gynecological tumor, comprising 4.8% of worldwide cancer incidence and 2.1% of mortality related to cancer [115,116]. Underlying risk factors are associated with high circulating levels of estrogen and include early menarche, obesity, diabetes mellitus, Lynch syndrome, nulliparity, late menopause, advanced age, breast cancer, tamoxifen therapy, and radiotherapy [117]. When considering both biological and clinical parameters, gene mutations are being used for EC classification [118]. EC can be divided into endometrioid (Type I), affecting approximately 80% of patients, and non-endometrioid (Type II) in the rest of the patients [119,120]. Non-endometrioid ECs include clear-cell carcinoma, endometrial serous carcinoma, and carcinosarcoma. Type 1 ECs have alterations in different genes, including *CTNNB1*, *PTEN*, *KRAS*, and DNA characterized by microsatellite instability (MSI) [121,122]. In contrast, Type 2 EC tumors are defined as having *TP53* mutations, amplification of *HER2*, increased *CDH1* expression, and a high Ki-67 (MIB1) score, which is a marker of proliferation. The standard treatment is total hysterectomy and bilateral salpingo-oophorectomy, which is usually effective for stage I disease [123]. However, in advanced stages, surgery is followed by radio- and/or chemotherapy. Despite advances in drugs and surgical treatments for EC, survival rates have not improved significantly. Thus, improving the ability to identify the prognostic risk factors of EC and formulating reasonable new treatment plans are essential for improving the prognosis and survival rate of patients with EC [124].

Vaginal and vulvar cancers are rare malignancies with similar estimated incidence and mortality rates [125]. Due to the difficulty in performing large prospective randomized trials in patients with these rare tumors, systemic chemotherapeutic regimens have generally been extrapolated from experience in the management of CC. This is because these malignancies share similar epidemiologic risk factors, are strongly associated with HPV infection and are predominantly of the squamous cell carcinoma (SCC) histologic subtype [126,127,128]. However, vaginal and vulvar cancers can be challenging to treat particularly when disease is not amenable to surgical resection or radiation [129]. There is currently no consensus on effective treatment as response rates to systemic chemotherapeutic regimens are variable in the recurrent setting for vaginal and vulvar SCC [129]. Additionally, given the tendency for vaginal and vulvar SCC to develop later in life, treatment options may be further limited by associated toxicity and co-morbidity [127,128]. Clinical and pathological prognostic factors are constantly being explored in order to minimize unnecessary treatments especially in elderly patients. Furthermore, new molecules are being investigated as targeted therapies to increase patient survival.

Building on the previous knowledge about the individual and combined effects of radiation and microgravity on cancer and tumor cell processes [130], the relatively few studies on GCs in simulated space conditions that have been published to date will be described.

### 5.2. Effects of Microgravity and Radiation on Gynecological Cancers: In Vitro and In Vivo Studies

Microgravity has different effects on normal and cancer cells, but the related underlying mechanisms are still being elucidated. Experiments using normal or cancer cells performed in space on the International Space Station (ISS) or under s-µg-conditions using devices approved by ESA and NASA to create µg conditions on Earth (known as ground-based facilities) [131], belong to a newly evolving area of research in oncobiology [132]. Numerous studies have demonstrated how a short- and long-term exposure to r- and s-µg influences differentiation, proliferation, migration, survival, apoptosis, adhesion, and other processes involved in carcinogenesis [53]. Space exploration missions also need to have strategies in order to mitigate the potentially harmful exposure to galactic cosmic radiation, which can cause cancer. Ongoing studies investigate these effects using cell- and animal-based studies in low Earth orbit [133]. The costs and logistic challenges involved when sending biological specimens to space have spearheaded the development of surrogate ground-based radiation experiments to study the mechanisms of biological injury and cancer risk. However, simulating galactic cosmic radiation has proven to be difficult. In fact, current studies are only partially succeeding at replicating the complexity of this radiation and its downstream injury pathways [133]. The following section discusses the few studies about various types of gynecological normal or cancer cells under conditions of weightlessness and/or real/simulated galactic cosmic radiation (Table 5 and Table 6). It is important to keep in mind that studies of cancer cells in culture do not necessarily reflect the actual tumor scenario in vivo.

#### 5.2.1. Ovarian Normal and Cancer Cells Exposed to Microgravity and/or Radiation

##### Altered Gravity and Microgravity

In order to study cellular interactions involved in the growth and differentiation of OC, a cell line, designated as LN1, was established from a mixed mullerian tumor of the ovary [134]. This cell line was cultured on microcarrier beads in the high aspect rotating-wall vessel (RWV), and the tumor cells readily proliferated without the need for cocultivation with a supportive cell layer. Phase contrast light microscopy and scanning electron microscopy showed the presence of three-dimensional multicellular aggregates consisting of multiple cell-coated beads bridged together, as well as scattered aggregates proliferating as spheroids free in suspension. This illustrates the ability of this culture system to provide the biological conditions necessary for pluripotent cell growth [134]. The same group later demonstrated that the RWV culture system is suitable for in vitro production of ovarian tumor cells with the same morphologic, oncogenic, and immunocytochemical characteristics shown in vivo [135].

In another study, LN1 cells were cultured for 14 days on the ISS during Expedition 3. When compared with ground controls, LN1 cells exposed to microgravity showed reduced expression of vimentin and epithelial membrane antigen [136], as well as reduced expression of IL-6 and IL-8 [132]. Both of these cytokines are associated with the growth of several types of tumors, including OC. Przystupski et al. showed that exposure to microgravity affects the morphology of SKOV-3 cells, as well as drug efficiency. Altered cell shape, presence of membrane blebbing and lamellipodia, and lack of filopodia have been observed in cells cultured on 3D-clinostat (3D-C). After exposure on the 3D-C with cisplatin, there was an increase in apoptotic cells and G0/G1 cell cycle arrest in comparison to the static control cells. Cell proliferation and migration were also altered after exposure to microgravity. These findings suggest that the altered gravity conditions affect cellular mechanisms that are involved in resistance to cisplatin. This is a crucial step towards understanding the relationship between cellular resistance to chemotherapy and the response to microgravity [137]. An integrated set of systems biology tools and databases were collated together by Mukhopadhyay et al., who analyzed more than 8000 molecular pathways on published global gene expression datasets of human cells in microgravity. Interestingly, microgravity alone may induce several cancer related signatures, including OC [138].

##### Radiation

In one particular experiment, six-week-old female B6C3F1 mice were exposed to 439 mGy heavy ion irradiation, as a 290 MeV/u carbon-ion beam (LET 10 keV/micron) at 2 cm from the upper proximal point of a spread Bragg beam, and were autopsied 13.5 months after the irradiation [139]. The total tumor incidence was 32.3% (mainly OC), in the irradiated group and 0% in the controls. These results indicate that heavy ion irradiation can induce ovarian tumors in females.

The effects of heavy ion and X-ray irradiation on tumorigenesis in B6C3F1 mice were investigated by exposure to 426 mGy heavy ion irradiation of 290 MeV/u carbon-ion beam (LET 60–210 KeV/micron) at the dose rate of 400 +/− 200 mGy/min, 500 mGy of X-ray irradiation at 100 mGy/min, or 5000 mGy of X-ray irradiation at 1000 mGy/min [140]. Interestingly, in the females after 13.5 months of whole-body irradiation, tumorigenicity was significantly lower for heavy ion than for 500 mGy and 5000 mGy X-ray irradiation. The incidences of OC (which was the main tumor), were 73%, 17%, and 41%, respectively. These findings indicate that 426 mGy of heavy ion irradiation is associated with a lower risk of inducing cancer than 5000 mGy of X-ray irradiation.

In another experiment carried out to investigate the relationship between oocyte apoptosis and ovarian tumors induced by high and low LET radiations, C57BL/6N mice were exposed to ^252^Cf fission neutron (2.13 MeV), 1000 mGy monoenergetic neutrons (0.317, 0.525, and 1.026 MeV), or ^137^Cs g-rays at 7 days of age [141]. The cumulative apoptotic index of oocytes was 65.6%, 77.9%, and 41.6% for the 2.13 MeV neutron, 0.525 MeV neutron, and g-rays, respectively. Follicles with apoptotic pregranulosa cells were 18.3%, 53.0%, and 22.8% of cumulative index for the three groups, respectively. Granulosa cell tumors developed only in the g-ray groups (3.2% for 1000 mGy and 15.6% for 3000 mGy), whereas tubular adenomas developed in the groups of g-ray (35.5%) and monoenergetic neutrons (26.1%). In addition, partial-body irradiation with 3000 mGy g-rays to the ovaries induced granulosa cell tumors in 27.3% of mice. These findings show that there is a higher effectiveness of neutrons than g-rays to induce oocyte and pregranulosa cell apoptosis, which correlates with the inhibition of the development of granulosa cell tumor.

Mishra et al. hypothesized that, in mice, charged iron particle irradiation induces ovarian carcinogenesis. Three-month-old female mice were exposed to 0 mGy (sham) or 500 mGy iron ions at the Brookhaven National laboratory and euthanized at 18 months. The 500 mGy irradiated mice showed signs of ovarian failure, with increased weight gain and lack of estrous cycling. A total of 7% and 47% of mice irradiated with 500 mGy had bilateral and unilateral ovarian tumors, respectively, whereas 14% of mice in the 0 mGy group had unilateral tumors. The tumors were tubular adenomas or mixed tubular adenoma/granulosa cell tumors. Though conclusions are limited by the dose rate, this study demonstrated a that space radiation analog can induce ovarian tumors in mice, raising concerns about ovarian tumors as late sequelae of deep space travel in female astronauts [142].

#### 5.2.2. Cervical Normal and Cancer Cells Exposed to Microgravity and/or Radiation

##### Microgravity

In a study investigating the multicellular interaction between CC cell lines and human umbilical vein endothelial cells (HUVEC), which were grown in a RWV, it was found that the co-culture presented tubular structures penetrating the tumor cell masses. This co-culture formed aggregates larger in size than the monocultures, with increased cell mass and number. This suggests that a RWV provides a new model that can be used to investigate the regulatory factors that govern tumor angiogenesis [143]. Kelly et al. investigated various cell types, including melanoma cells, prostate cancer cells, osteosarcoma cells, lung cancer cells, and cervical carcinoma cells (HeLa) on the NASA-developed hydrofocusing bioreactor (HFB) and the rotary cell culture system (RCCS). It has been demonstrated that HFB exposure increased CD133-positive cell growth from various cell lines, when compared with the RCCS vessel and normal gravity control [144].

##### Spaceflight Studies

So far, the majority of studies on spaceflight focus on normal cells and tissues. However, little is known of the effects of spaceflight on cancer cells. To investigate the potential effects of the exposure of the space environment on cancer cells, one of the experiments included sending human cervical carcinoma CaSki cells on “Shen Zhou IV” space shuttle mission. The cell morphology and proliferation were investigated after flying to ground. The growth of CaSki cells in the flight group was slow when compared with ground groups. Light microscopy revealed differences in cell morphology between ground controls and flight groups, with the latter being characterized by smaller, rounder, smoother and low adhesion cells. Furthermore, space-grown CaSki cells showed altered gene expression in genes regulating the cell cycle, cell morphology, signal transduction, and apoptosis [145,146].

##### Radiation

In contrast to the numerous studies that have been conducted on the ground using particles generated in accelerators, investigations on DNA damage from direct exposure to natural space radiation are very limited. In one study, where fixed human cervical carcinoma (HeLa) cells were flown in the Russian MIR space station for 40 days or on the Space Shuttle for 9 days, the resulting DNA damage levels, as measured by enzymatic incorporation of [3 H]-dATP from terminal deoxyribo-nucleotidyl transferase, correlated with the space flight duration. This suggests that the measured DNA damage was caused by space radiation and was dependent on the duration of the space flight [71]. However, further experiments need to be performed in the true space environment in order to further investigate and address this critical question.

##### Viral Reactivation

A potential inducer of gynecological cancers during space travel may be linked to oncogenic virus reactivation. It has been reported that HPV is responsible for 4.5% of CCs and 630,000 new cancer cases per year [147]. HPV can infect both genders and can also cause anal, penis, vagina, vulva, and oropharynx cancers. Despite immune clearance, some viral infections may persist in the latent phase and can cause reactivation or outbreaks. HPV virus latency and reactivation has been widely documented in literature [148], and this reactivation risk increases in women with co-infections, such as HIV and herpesviruses [149]. HPV and co-infection with HSV-2 have been reported in both cervical precancerous lesions and invasive CCs with a prevalence of 13–30% [149]. There is also evidence of the Epstein–Barr virus (EBV), human herpesvirus 4, as a cofactor in cervical pathologies. EBV has been detected in CIN and CC cells [149].

In 2017, Mehta et al. reported reactivation of latent EBV, varicella-zoster virus, and cytomegalovirus in a population of astronauts (male and female) as well as increased in viral copy numbers during long-duration space travel in comparison to short-duration space missions (10 to 16 days) [150]. During spaceflight, immune dysregulation, impaired NK cell function, and reduced T cell activation have all been reported [151,152]. Both short- and long-duration spaceflights can cause reactivation of the latent herpes virus infections [153]. In addition to the herpes virus, varicella zoster virus (VZV), cytomegalovirus (CMV), and EBV shedding increased in ISS missions [153].

Currently, it is unknown if spaceflight would alter the HPV clearance and/or reactivation. Based on the current evidence of immune dysfunction and reactivation of some viruses, HPV screening may need to be modified in the future, especially for longer duration spaceflights. Up to 23% of astronaut candidates had a history of treated or current cervical dysplasia. If research shows increased risk for reactivation of viruses like HPV in spaceflight, these women may be at increased risk for CC [154].

#### 5.2.3. Endometrial Normal and Cancer Cells Exposed to Microgravity and/or Radiation

##### Microgravity

The possible effect of simulated microgravity (SM) on the process of proliferation and in vitro decidualization was investigated in primary human endometrial stromal cells (eSCs) (Cho et al.). Following 36 h of exposure to SM, there was a decrease in the proliferation and migration of eSCs, without inducing apoptosis and changes in cell cycle progression. A decrease in the phosphorylation of Akt and levels of matrix metalloproteinase (MMP)-2 and FOXO3a were also observed, impeding autophagic flux by reducing the levels of autophagy-related genes. Overall, these results suggest that exposure to SM decreases proliferation and migration in eSCs through Akt/MMP and FOXO3a/autophagic flux [155].

A three-dimensional (3D) cell culture model of human EC was established by Grun et al., using a RCCS and appeared histologically similar to the primary tumors. This is likely to be useful in the study of the molecular and biological mechanisms of endometrial tumor progression and especially when testing novel molecular targets for cancer therapy [156].

##### Radiation

The effect of space radiation on expression of apoptosis-related genes was investigated in endometrial cells (HEC1B and AN3CA cells) by Palumbo et al., whereby cell death was induced by monoenergetic protons (1000–10,000 mGy; LET 8.35 keV/μm and 4.86 MeV) and γ-rays (200–1600 mGy) after irradiation for 4 h. Following exposure to 1000 mGy protons and 400 mGy γ-rays, HEC1B cells underwent apoptosis, as assessed by presence of PARP cleavage. However, this was not observed after higher doses, as cells were likely to have progressed directly to necrosis. AN3CA cells, which are less differentiated than HEC1B, did not undergo apoptosis, but underwent rapid necrosis following 10,000 mGy proton radiation and above 200 mGy γ-radiation. Since the experiments included only three replicates per group, with no statistical analyses, it is still unclear whether there is greater potency of proton radiation compared to γ-radiation for these endpoints in uterine carcinoma cells [157].

**Table 5:** Studies on effects of space flight and simulated gravity on gynecological tissues.

Tissue Type	Microgravity/Space Flight	Cell/Animal Models	Effect	Study
Ovarian	simulated microgravity RWV	LN1 human ovarian tumor cells	LN1 cells grew as spheroids free in suspension	Becker et al., 1993; Goodwin et al., 1997 [134,135]
	spaceflight (cells were cultured on the ISS)	LN1 human ovarian tumor cells	Cells showed reduced expression of VIM and EMA	Hammond et al., 2005 [136]
	simulated microgravity 3D-C	SKOV-3 human ovarian cancer cells	Cells showed reduced proliferation, migration, and higher sensitivity of cancer cells to the cisplatin	Przystupski et al., 2021 [137]
	microgravity	set of systems-biology tools and databases	identified several cancer related signatures induced by microgravity	Mukhopadhyay et al., 2016 [138]
Cervical	simulated microgravity RWV	Co-culture of HUVEC and tumor primary cells	Co-culture presented tubular structures penetrating the tumor cell masses,	Chopra et al., 1997 [143]
	simulated microgravity HFB and RCCS	HeLa human cervical cancer cells	HFB exposure increased CD133-positive cell growth	Kelly et al., 2010 [144]
	spaceflight (cells were flown on “Shen Zhou IV” space shuttle mission)	Human cervical carcinoma CaSki cells	Cells exhibited morphologic differences, characterized by rounder, smoother, decreased, smaller, and low adhesion cells. Furthermore, space-grown cells showed altered gene expression that generally corresponded to changes in genes regulating the cell cycle, cell morphology, apoptosis, and signal transduction	Zhang et al., 2011; Guo et al., 2012 [145,146]
Endometrial	simulated microgravity 3D-C	human endometrial stromal cells (eSCs)	Cells showed reduced proliferation and migration. This was accompanied by a simultaneous decrease in the phosphorylation of Akt and the level of matrix metalloproteinase (MMP)-2 and FOXO3a.	Cho et al., 2019 [156]
	simulated microgravity RCCS	Human tumor primary cells	3D model endometrial cancer cell culture was established	Grun et al., 2009 [157]

Abbreviations: RWV: rotating-wall vessel; ISS: International Space Station; 3D-C: 3D-clinostat; VIM: vimentin; EMA; epithelial membrane antigen; HUVEC: human umbilical vein endothelial cells; HFB: hydrodynamic focusing bioreactor; RCCS: rotatory cell culture system.

**Table 6:** Studies on effects of irradiation on gynecological tissues.

Tissue Type	Radiation Type	Cell/Animal Models	Effect	Study
Ovarian	0.439 Gy as a 290 MeV/u carbon-ion beam (LET 10 keV/micron)	B6C3F1 mice	Induction of ovarian tumors	Watanabe et al., 1998 [139]
	0.426 Gy heavy ion irradiation of 290 MeV/u carbon-ion beam (LET 60–210 KeV/micron) at the dose rate of 0.4 +/− 0.2 Gy/min; 0.5 Gy of X-ray irradiation at 0.1 Gy/min or 5 Gy of X-ray irradiation at 1 Gy/min.	B6C3F1 mice	Tumorigenicity was lower for heavy ion than for 0.5 Gy and 5 Gy X-ray irradiation	Watanabe et al., 1998 [140]
	high and low LET radiations. 1.0 Gy monoenergetic neutrons (0.317, 0.525 and 1.026 MeV), 252Cf fission neutron (2.13 MeV) or 137Cs γ-rays	C57BL/6N mice	Higher effectiveness of neutrons than γ-rays to induce oocyte and pregranulosa cell apoptosis correlates with the inhibition of granulosa cell tumor development	Nitta & Hoshi, 2003 [141]
	HZE particles. 50 cGy iron ions	C57BL/6J	Induction of ovarian tumors	Mishra et al., 2018 [142]
Cervical	spaceflight (cells were flown on “Russian MIR” space station or on the Space Shuttle)	HeLa human cervical cancer cells	Increased DNA damage	Ohnishi, et al., 2002 [71]
Endometrial	Monoenergetic protons (1–10 Gy; LET 8.35 keV/μm and 4.86 MeV) and γ-rays (0.2–1.6 Gy)	Human endometrial carcinoma cell lines (HEC1B and AN3CA cells)	Decreased cell survival	Palumbo et al., 2001 [158]

Abbreviations: LET: linear energy transfer; HZE: high-charge and energy.

## 6. Current Challenges in Gynecological Cancer Risk Prediction for Spaceflight

Currently, there is no evidence to suggest that female astronauts have an increased incidence of gynecological cancers. However, we should note that given the low number of female astronauts, conducting studies to determine whether spaceflight increases gynecological cancer risk is difficult. Another possibility could be that the current limitations enforced on the time females spend in space are effective at reducing the incidence of gynecology-specific cancers. At present, studies focusing on subjects that experience similar occupational risk factors to astronauts is the closest form of data we can use to address cancer risk in female astronauts. For example, there are epidemiological studies that have found that there is an increased incidence of breast cancer in female commercial flight attendants. Since female astronauts are exposed to similar occupational risk factors and may also have piloting experience, they may also be at an increased risk for breast cancer [15].

### 6.1. Gynecologic Medical Standards for Career and Private Astronauts

The prevention of gynecologic morbidity in space begins with the selection process and continues with personalized preventive medicine programs during the astronaut’s Earth-based career [158]. The medical selection criteria for female astronauts with different space agencies undertaking short- and long-term journeys in LEO are identical to those of males except for reproductive system standards and radiation exposure limits. Until 2022, career exposure limits for women of all ages were lower than those for men [14]. The difference in radiation exposure limits reflected the increased incidence of breast, thyroid, and OC in women compared to the incidence in men and the increased risk of lung cancer among female atomic bomb survivors. Moreover, due to reduced cardiovascular and trauma risks, women live approximately 5–7 years longer than men, thus allowing for more time for post flight radiation-induced carcinogenesis. However, the new radiation standard for the radiation exposure limit is now set to be less than 600 mGy and is universal for all ages and sexes. For example, the updated value has been fully integrated into the NASA Space Flight Human-System Standard on Crew Health that sets standards for fitness for duty, space permissible exposure limits, and permissible outcome limits, as well as levels of medical care, medical diagnosis, intervention, treatment and care, and countermeasures [159].

Gynecologic selection standards for astronauts have evolved and generally have been relaxed as spaceflight experiences progress [158]. Current medical standards allow for a history of endometriosis but would disqualify candidates with endometriosis that results in severe dysmenorrhea, endometriomas, or extensive pelvic adhesive diseases. Premenstrual syndrome must interfere with performance of duties to disqualify a female candidate during selection. Any gynecologic malignancy is disqualifying for selection and for flight except for successfully treated cervical carcinoma in situ. As part of the final astronaut selection process, each female candidate finalist undergoes pelvic and abdominal sonography, colposcopy, gynecologic examination, pap smear, and screening for high-risk HPV. Up to the 2013 selection, no female finalists have been disqualified because of gynecologic conditions found at the time of the selection examination. However, several female astronaut finalists were required to undergo surgical procedures or biopsies to rule out disqualifying pathology or neoplasia in ovarian masses, breast masses, or breast microcalcifications, or to remove large leiomyomata uteri [158].

For astronauts, the annual examination includes a physical test by a flight surgeon. Extensive blood analysis, periodic exercise capacity tests, mammography or breast MRI, and bone density analysis (every 3 years unless postflight). Colposcopy is performed based on current practice guidelines and known individual risk factors. During the examination, careful consideration is given to effective contraception for training and flight, optimizing bone density, potential pregnancy timing, and desire for menstrual control during an upcoming mission. For astronauts over 35 years of age and experiencing spotting or breakthrough bleeding on cyclic or combined oral contraceptive, a saline infusion sonogram or hysteroscopy is usually completed to rule endometrial polyps, submucous myomas, or other abnormalities of the endometrial cavity.

Preflight medical evaluations are more comprehensive than annual exams and include abdominal and pelvic ultrasound studies and breast MRI. Currently, all female astronauts receive a pre-flight transvaginal ultrasound (TVUS). While TVUS is not recommended for routine terrestrial OC screening, surgical management of ovarian masses can be considered [16]. Furthermore, if an endometrial stripe abnormality is noted on TVUS, terrestrial guidelines for endometrial hyperplasia/cancer screening can be considered, especially in the setting of abnormal uterine bleeding [160]. In addition to the CC screening recommended by the American Society for Colposcopy and Cervical Pathology (ASCCP), all astronauts should be encouraged to obtain the HPV vaccine series. Routine screening for a personal and family history of precancerous lesions and cancer is recommended to trigger consideration for preflight genetic screening of hereditary cancer syndromes. Lastly, when discussing the risks and benefits of hormonal modalities during spaceflight, counseling regarding the known effect on breast, ovarian, and EC should be part of this counseling.

Astronauts in training that develop a disqualifying gynecologic condition are often granted a waiver if the medical threat or risk can be eliminated or reduced to an accepted level or the condition, so that it does not interfere with performance of duties or mission assurance. Decisions regarding waivers for flight to the ISS are made on a case-by-case basis by NASA and the International Partners following a thorough review of the condition, inputs from specialist consultants, successful therapeutic intervention (if needed), and complete recovery. Final determination of medical certification currently rests with NASA and the Multilateral Space Medicine Board of the ISS International Partners (MSMBIP). For the postflight female astronaut population, more conservative recommendations including annual mammography with adjunctive ultrasound for dense breast tissue, or alternating mammography with biennial breast magnetic resonance imaging (MRI), earlier and more frequent colon cancer screening (starting at 40 y and every 5 y thereafter), and an annual skin exam by dermatologist [154,158] have been recommended [161].

In the case of future female spaceflight participants on NASA-sponsored commercial crew orbital flights (Boeing and SpaceX), Axiom missions, and Inspiration 4 missions, they will probably come under standards and medical selection testing that evolved from a previously published ISS Medical Evaluation Document Volume C and Appendix F [162]. The published standard finds certain gynecologic conditions disqualifying, and individuals outside the standards can be assessed for a waiver based on a risk assessment/mitigation approach. Disqualifying conditions include: (1) disease, injury, or other disorders of the gynecologic tract that could require emergency treatment or interfere with mission completion; (2) any disabling disorders of the reproductive system or associated anatomical structures that could potentially require emergency medical care; and/or (3) history of tumors or pathological growth will be reviewed by the MSMBIP. However, totally private spaceflight participants traveling to LEO or on suborbital flights will have limited flight-related responsibilities and come under the jurisdiction of the US Federal Aviation Administration and each company’s medical policy. Proposed gynecology guidance for private spaceflight participants includes assessing the history of surgery, medication use, current pregnancy, recent postpartum state, or recent pregnancy loss status [158].

### 6.2. Countermeasures

There are numerous protective measures specific to ionizing space radiation exposure that could be considered, particularly for exploration-class missions. As discussed, space radiation remains one of the primary factors limiting human tolerance to long-term spaceflight. At present, one of the principal countermeasures to protect astronauts from the biological effects of space radiation is limiting the time spent in space. However, this is not feasible for long-term interplanetary space travel, so other remaining measures will have to be implemented.

Preventing space radiation exposure through shielding remains a major challenge for space travel. Reasons for this include restrictions on cost, spacecraft mass, and the nature of ionizing particles, which can penetrate spacecraft hulls and result in secondary intra-vehicular radiation [19]. Spacecraft shielding can be divided into two main categories: passive shielding, constructed of specific materials and always present on spacecraft, and active shielding, which utilizes magnetic or electrostatic fields. In 2019, Barthel and Sarigul-Klijn reviewed shielding optimization methods for space travel beyond the influence of the Earth’s magnetic fields [163].

Regarding passive shielding, optimization of shielding placement could help overcome the mass limitations of spacecraft. Furthermore, due to the interactions between the spacecraft and ionizing particles, materials with lighter nuclei atoms are ideal as high mass nuclei in shielding materials would increase the number of neutrons inside the spacecraft; an electron plasma would be most effective followed by liquid hydrogen. Special space suits with built-in shielding have also been proposed to eradicate the cost of shielding an entire spacecraft but to still provide reasonable protection to astronauts. Active shielding could provide protection, potentially without compromising the mass of spacecraft to the same degree. Electrostatic shielding would work by placing several charged spheres in specific orientations around the spacecraft. This would create a “safe zone” in a particular location on the spacecraft. Magnetic shielding would be created by using a superconducting solenoid around the spacecraft, generating a high magnetic field that would deflect particles below a certain energy threshold. The shielding technology described still has a long way to go in its development but holds the potential to provide effective protection to astronauts on exploration class missions.

Biological countermeasures could also be utilized to protect against the ill-effects of ISR-induced oxidative stress. In 2021, Montesinos et al. discussed the role of these countermeasures, which will also be summarized here [164]. Several studies have found that dietary measure and supplementation is likely to have a protective effect in astronauts. It has long been known that a varied diet full of plant foods provides us with the nutrition needed for optimum health. The limitations of storage space and mass on spacecraft restricts the access of crew to a wide variety of foods that would provide them with an abundance of phytochemicals, antioxidants, and other compounds known to interfere with oxidative stress pathways.

*N*-Acetyl-l-Cysteine (NAC) contains an acetyl group that enhances its lipophilicity and allows for the molecule to cross the cell lipid bilayer. This contributes to replenishment of glutathione and thus may reduce both cellular ROS and mitochondrial damage. Because NAC tends to be more stable, it can be stored and administered in the form of a supplement to enhance cellular antioxidant capacity to combat the adverse effects of space environment exposure during space travels. DNA protection was observed in mice who received oral NAC treatment followed by a whole-body irradiation of 1000 mGy gamma radiation at a dose rate of 5000 mGy/min [165]. Treatment with NAC increased the overall health and quality of post ovulatory oocytes in vitro. Reduced spindle defects, decreased abnormal mitochondrial distribution, reduced reactive oxygen species, increased levels of intracellular ATP, and decreased abnormal cortical granules distribution were observed in oocytes treated with NAC [166]. However, more studies are needed to try to closely replicate space radiation to more accurately determine the effects of such radiation or the protective effects of countermeasures such as this.

Selenium is amongst the group of micronutrients that is found to be depleted during long-duration spaceflight. This compound is needed for the normal function of oxidative stress protection molecules like glutathione peroxidase, thioredoxin reductase, and the selenoprotein family [167]. Selenium deficiency is also associated with dysfunction of the immune system [168]. Furthermore, taurine is a sulfur-containing amino acid also involved in mitochondrial health. Previous studies suggested that taurine conjugates with tRNALeu (UUR) or tRNALys (UUU) of mitochondria, which is important for proper codon-anticodon matching, thus increasing the accuracy and quality of mitochondrial proteins produced [169]. Supplementation of selenium, taurine, and other compounds that protect against oxidative stress might be important in lessening the ionizing effects of long-term radiation exposure on interplanetary space travel. However, many antioxidant compounds are typically needed in large doses in order to achieve the desired clinical effect as single agents. Unfortunately, the doses needed to achieve this exceed tolerability and/or safe levels. The use of a single agent may also downregulate other natural antioxidant pathways, given the complexity of these mechanisms in the human body [170]. Due to the limitations on the dietary intake of astronauts, the development of a single supplementation method that combines safe levels of the most effective chemopreventive compounds would be most desirable for long-term spaceflight missions. Better yet, developing a way for astronauts to grow their own fresh produce in space from which they can source their antioxidants and nutrients would be another desirable way of providing astronauts with compounds that are protective against oxidative stress and cancer development. Other studies have looked into several interesting biological methods like injecting tardigrade DNA [171] or neulasta, which is a bone marrow stimulant, into human tissue [172]. When injected in mini pigs, these were found to have a modest effect on the body’s response to irradiation.

With respect to women astronauts, hormonal contraception is already standard practice, which may have countermeasure impacts. There are also non-contraceptive health benefits of using hormonal contraceptives. Many considerations related to cancer risk have been examined on Earth. There are extensive data demonstrating combined oral contraceptive use protects against the development of colon, endometrial, and OC, with increasing protective effects with longer duration use [173]. Similarly, the levonorgestrel intrauterine device is associated with a reduced risk of EC and OC [173]. However, CC risk has been found to increase with increasing duration of oral contraceptive use and newer combined hormonal contraceptive options of the vaginal ring and transdermal patch have a similar increased risk of CC [174,175]. Progestin-only contraceptive users do not have an increased relative risk of developing CC [175]. Furthermore, in HPV-positive women, it has been suggested that the estrogens and progestogens in hormonal contraception could enhance expression of HPV genes through hormone response elements on the viral genome, consequently increasing the risk of CC [176].

Current combined oral contraceptive use is associated with a slightly higher risk of breast cancer (7%); however, this appears to be more associated with triphasic oral contraceptive pills and decreases after discontinuation of oral contraceptives [177,178]. Current evidence also suggests that levonorgestrel intrauterine device users also have an increased risk of developing breast cancer, and this risk is increased with increasing age [178,179]. Overall, the benefits of hormonal contraceptive use outweigh the slight increased risks of cancer development. This information is reassuring, however, as mentioned before, there is a lack of knowledge on hormonal contraception use for long-term spaceflight missions, and contraception use is still an important factor to account for in the overall reproductive health of female astronauts. The risk of breast cancer in female astronauts also merits further study, especially with relation to the exposure to space radiation.

Additionally, proposed pharmacological measures include, but are not limited to, pharmaceutical radioprotectors and immunomodulation. Pharmaceutical radioprotectors are administered before exposure to a radiation environment. Radiation mitigators are administered after exposure but before the signs and symptoms of radiation exposure occur [180]. To date, the use of these agents thus far in controlled clinical scenarios, where radiation exposure can be controlled and quantitated, does not reflect the unpredictable radiation environment in space. Timing the administration of these agents would therefore be much harder in astronauts [164]. Immunomodulation-based agents include prospective DNA- and RNA-based anti-radiation vaccines in addition to anti-radiation antidotes. These would work via a group of antibodies and natural inhibitory proteins that prevent inflammation, pathological apoptosis, and necrosis of radiation-exposed tissues [164].

As discussed before, radiation has been shown to have molecular effects. These include genetic and epigenetic changes that alter molecular functioning and can lead to carcinogenesis [181]. Genetic counseling and screening guidelines are available through the American College of Obstetricians and Gynecologists (ACOG), the American College of Medical Genetics and Genomics (ACMG), National Society of Genetic Counselors (NSGC), and the Society of Gynecologic Oncology (SGO), and it is reasonable to consider screening astronauts preflight for genetic risk factors for certain cancers. Consideration may need to given for genetic screening, especially of female astronauts for certain mutations such as BRCA1/BRCA2 and others associated with breast cancer. If a carrier condition has been identified, screening and risk-reduction protocols can be discussed depending on the type of mutation. Terrestrially, risk-reduction surgeries depending on the mutation recommended at various ages. Salpingo-oophorectomy is recommended at age 35–40 years for BRCA1 carriers and at age 40–45 years for BRCA2 carriers. For BRIP1, RAD51C, and RAD51D carriers, it is recommended at age 45–50 years [182].

While work is ongoing in categorizing the radiosensitive/radioresistant genes, we should note that this may be an interesting application of gene/epigenetic modification technologies as radiation countermeasures. Recent advances in these methods have shown improved overall outcomes, where approaches altering the epigenome are readily reversible [183,184]. Successful identification and modulation of these genes could allow for a significant reduction in negative outcomes experienced by future generations of astronauts.

## 7. Future Perspectives

Omics approaches are likely to help in understanding the human health risks due to spaceflight missions. Since its inception in 2014, the NASA GeneLab data repository currently boasts over 271 omics data collected from spaceflight and ground simulation experiments, but it contains no data on female human gynecological cancers [185]. Interestingly, there exists only one dataset relevant to females, and the study only investigated the mammary gland of mice [186]. Therefore, there is a significant need for studies that investigate female physiology in space.

There are a number of research opportunities that remain for considering the risks and management of breast and gynecologic cancers pre- and postflight. These include the following: histological differences of any tumors that have occurred in astronauts, characterizing molecular pathways involved in carcinogenesis in analog mammalian models, exploratory omics endpoints, the possibility HPV reactivation, the role of radiation countermeasures in prevention, optimizing in-flight or postflight screening protocols, changes in the microbiota of the reproductive tract during spaceflight and their relation to gynecological cancers, and evaluating the role that artificial intelligence and machine learning will play in cancer detection and prevention. Further study on gynecological cancers, including the incidence of the rare types, in space will build a knowledge base that will help to ensure the safety and health of female astronauts by giving them access to prevention, diagnosis, and countermeasures during their space missions.

It would be extremely beneficial to the community to come with a roadmap of studies that needs to be conducted so gynecological risks associated with exploration class missions to the Moon and Mars are minimized. Further, as commercial and private astronauts become more common, obtaining gynecologic and reproductive endpoints following spaceflight will be critical for the creation of preflight, in-flight, and postflight protocols for the screening, diagnosis, and management of gynecologic morbidity and overall astronaut safety.

## 8. Conclusions

The current data reveal a scarcity of knowledge about the impact of space radiation and microgravity on gynecologic cancer, as there have been insufficient numbers of female astronauts exposed to long-duration, low-dose rate, and proton and heavy ion radiation to reliably determine the impact on the female reproductive system. With the upcoming Artemis missions set to place the first females on the Moon, there will be longer duration of exposures to both microgravity and space radiation; hence, the influence of flight length on risks related to gynecological cancers will demand a larger focus in ensuring astronaut safety during flight and postflight. Indeed, among all body tissues, the male and female gonads are among the most sensitive to radiation. Therefore, these tissues may be one of the best to study to determine space radiation related exposures.

## Figures and Tables

**Table 1:** Types of radiation and the radiation weighting factor.

Type of Radiation	Radiation Weighting Factor (WR)
X-rays/Gamma rays	1
Electrons	1
Protons	2–5
Neutrons	5–20
Heavy ions	20

**Table 2:** Radiation effects on female reproductive function.

Age	Dose, mGy	Effect
All ages	1700	Temporary sterility lasting 1–3 years
	1250–1500	Amenorrhea in 50%
	3200–6250	Permanent sterility
Ages 15–40	1250–2500	Temporary amenorrhea
	2500–5000	Ovulary suppression in 40–100%
	5000–8000	Permanent ovulary suppression in 40–100%
	8000–20,000	Permanent ovulary suppression in 100%

## Data Availability

This review paper does not report any new data.

## Abbreviations

ACMG	American College of Medical Genetics and Genomics
ACOG	American College of Obstetricians and Gynecologists
ASCCP	American Society for Colposcopy and Cervical Pathology
CC	cervical cancer
CSA	Canadian Space Agency
3D-C	3D-clinostat
EC	endometrial cancer
ESA	European Space Agency
GCR	galactic cosmic radiation
HFB	hydrofocusing bioreactor
HPV	human papillomavirus
HZE	high Z and energy
ISS	International Space Station
JAXA	Japan Aerospace Exploration Agency
LEO	low Earth orbit
LET	Linear Energy Transfer
MSMBIP	Multilateral Space Medicine Board of the ISS International Partners
NAC	N-Acetyl-L-Cysteine
NASA	National Aeronautics and Space Administration
NSGC	National Society of Genetic Counselors
OC	ovarian cancer
RBE	relative biological effectiveness
RCCS	rotary cell culture system
RER	radiation effects ratio
RWV	rotating-wall vessel
SGO	Society of Gynecologic Oncology
SPE	solar particle events

## References

[1] Steller J.G., Alberts J.R., Ronca A.E. (2018). Oxidative Stress as Cause, Consequence, or Biomarker of Altered Female Reproduction and Development in the Space Environment. Int. J. Mol. Sci..

[2] Cortés-Sánchez J.L., Callant J., Krüger M., Sahana J., Kraus A., Baselet B., Infanger M., Baatout S., Grimm D. (2021). Cancer Studies under Space Conditions: Finding Answers Abroad. Biomedicines.

[3] Afshinnekoo E., Scott R.T., MacKay M.J., Pariset E., Cekanaviciute E., Barker R., Gilroy S., Hassane D., Smith S.M., Zwart S.R. (2020). Fundamental Biological Features of Spaceflight: Advancing the Field to Enable Deep-Space Exploration. Cell.

[4] Axpe E., Chan D., Abegaz M.F., Schreurs A.S., Alwood J.S., Globus R.K., Appel E.A. (2020). A human mission to Mars: Predicting the bone mineral density loss of astronauts. PLoS ONE.

[5] Patel Z.S., Brunstetter T.J., Tarver W.J., Whitmire A.M., Zwart S.R., Smith S.M., Huff J.L. (2020). Red risks for a journey to the red planet: The highest priority human health risks for a mission to Mars. NPJ Microgravity.

[6] Crucian B., Simpson R.J., Mehta S., Stowe R., Chouker A., Hwang S.A., Actor J.K., Salam A.P., Pierson D., Sams C. (2014). Terrestrial stress analogs for spaceflight associated immune system dysregulation. Brain Behav. Immun..

[7] Leser N., Wagner S. (2015). The effects of acute social isolation on long-term social recognition memory. Neurobiol. Learn. Mem..

[8] Reschke M.F., Cohen H.S., Cerisano J.M., Clayton J.A., Cromwell R., Danielson R.W., Hwang E.Y., Tingen C., Allen J.R., Tomko D.L. (2014). Effects of sex and gender on adaptation to space: Neurosensory systems. J. Women’s Health.

[9] Clark B.C., Fernhall B., Ploutz-Snyder L.L. (2006). Adaptations in human neuromuscular function following prolonged unweighting: I. Skeletal muscle contractile properties and applied ischemia efficacy. J. Appl. Physiol..

[10] Ronca A.E., Baker E.S., Bavendam T.G., Beck K.D., Miller V.M., Tash J.S., Jenkins M. (2014). Effects of sex and gender on adaptations to space: Reproductive health. J. Women’s Health.

[11] Newhauser W.D., Zhang R. (2015). The physics of proton therapy. Phys. Med. Biol..

[12] RCR—Royal College of Radiologists (2019). Radiotherapy Dose Fractionation Third Edition. https://www.rcr.ac.uk/publication/radiotherapy-dose-fractionation-third-edition.

[13] Freese S., Reddy A.P., Lehnhardt K. (2016). Radiation impacts on human health during spaceflight beyond Low Earth Orbit. REACH.

[14] Jones C.B., Mange A., Granata L., Johnson B., Hienz R.D., Davis C.M. (2019). Short and Long-Term Changes in Social Odor Recognition and Plasma Cytokine Levels Following Oxygen (16O) Ion Radiation Exposure. Int. J. Mol. Sci..

[15] Barr Y.R., Bacal K., Jones J.A., Hamilton D.R. (2007). Breast cancer and spaceflight: Risk and management. Aviat. Space Environ. Med..

[16] Steller J.G., Blue R.S., Burns R., Bayuse T., Antonsen E.L., Jain V., Blackwell M.M., Jennings R.T. (2020). Gynecologic Risk Mitigation Considerations for Long-Duration Spaceflight. Aerosp. Med. Hum. Perform..

[17] Zeitlin C., Hassler D.M., Cucinotta F.A., Ehresmann B., Wimmer-Schweingruber R.F., Brinza D.E., Kang S., Weigle G., Böttcher S., Böhm E. (2013). Measurements of energetic particle radiation in transit to Mars on the Mars Science Laboratory. Science.

[18] Wilson J.W. Environmental geophysics and SPS shielding. Proceedings of the Workshop on the Radiation Environment of the Satellite Power System.

[19] Chancellor J.C., Scott G.B., Sutton J.P. (2014). Space Radiation: The Number One Risk to Astronaut Health beyond Low Earth Orbit. Life.

[20] Simpson J.A. (1983). Elemental and isotopic composition of the galactic cosmic rays. Ann. Rev. Nucl. Part. Sci..

[21] Ackermann M., Ajello M., Allafort A., Baldini L., Ballet J., Barbiellini G., Baring M.G., Bastieri D., Bechtol K., Bellazzini R. (2013). Detection of the characteristic pion-decay signature in supernova remnants. Science.

[22] Cucinotta F.A., Durante M. (2006). Cancer risk from exposure to galactic cosmic rays: Implications for space exploration by human beings. Lancet Oncol..

[23] Onorato G., Di Schiavi E., Di Cunto F. (2020). Understanding the Effects of Deep Space Radiation on Nervous System: The Role of Genetically Tractable Experimental Models. Front. Phys..

[24] Dymova M., Dmitrieva M., Kuligina E., Richter V., Savinov S., Shchudlo I., Sycheva T., Taskaeva I., Taskaev S. (2021). Method of Measuring High-LET Particles Dose. Radiat. Res..

[25] Cortese F., Klokov D., Osipov A., Stefaniak J., Moskalev A., Schastnaya J., Cantor C., Aliper A., Mamoshina P., Ushakov I. (2018). Vive la radiorésistance!: Converging research in radiobiology and biogerontology to enhance human radioresistance for deep space exploration and colonization. Oncotarget.

[26] Tommasino F., Durante M. (2015). Proton radiobiology. Cancers.

[27] Saha J., Wilson P., Thieberger P., Lowenstein D., Wang M., Cucinotta F.A. (2014). Biological characterization of low-energy ions with high-energy deposition on human cells. Radiat. Res..

[28] Benton E.R., Benton E.V. (2001). Space radiation dosimetry in low-Earth orbit and beyond. Nucl. Instrum. Methods Phys. Res. B..

[29] International Commission on Radiological Protection (1991). 1990 Recommendations of the International Commission on Radiological Protection. Annals of the ICRP.

[30] Chancellor J.C., Blue R.S., Cengel K.A., Auñón-Chancellor S.M., Rubins K.H., Katzgraber H.G., Kennedy A.R. (2018). Limitations in predicting the space radiation health risk for exploration astronauts. NPJ Microgravity.

[31] Schwadron N.A., Blake J.B., Case A.W., Joyce C.J., Kasper J., Mazur J., Petro N., Quinn M., Porter J.A., Smith C.W. (2014). Does the worsening galactic cosmic radiation environment observed by CRaTER preclude future manned deep space exploration?. Space Weather.

[32] Papaioannou A., Anastasiadis A., Sandberg I., Jiggens P. (2018). Nowcasting of Solar Energetic Particle Events using near real-time Coronal Mass Ejection characteristics in the framework of the FORSPEF tool. J. Space Weather Space Clim..

[33] Restier-Verlet J., El-Nachef L., Ferlazzo M.L., Al-Choboq J., Granzotto A., Bouchet A., Foray N. (2021). Radiation on Earth or in Space: What Does It Change?. Int. J. Mol. Sci..

[34] Zeitlin C., Hassler D.M., Ehresmann B., Rafkin S.C.R., Guo J., Wimmer-Schweingruber R.F., Berger T., Matthiä D. (2019). Measurements of radiation quality factor on Mars with the Mars Science Laboratory Radiation Assessment Detector. Life Sci. Space Res..

[35] Mishra B., Luderer U. (2019). Reproductive hazards of space travel in women and men. Nat. Rev. Endocrinol..

[36] Serova L.V., Denisova L.A. (1982). The effect of weightlessness on the reproductive function of mammals. Physiologist.

[37] Hong X., Ratri A., Choi S.Y., Tash J.S., Ronca A.E., Alwood J.S., Christenson L.K. (2021). Effects of spaceflight aboard the International Space Station on mouse estrous cycle and ovarian gene expression. NPJ Microgravity.

[38] Lei X., Cao Y., Ma B., Zhang Y., Ning L., Qian J., Zhang L., Qu Y., Zhang T., Li D. (2020). Development of mouse preimplantation embryos in space. Natl. Sci. Rev..

[39] Alberts J.R., Ronca A.E. (2005). Development as adaptation: A paradigm for gravitational and space biology. Adv. Space Biol. Med..

[40] Ronca A.E., Alberts J.R. (2000). Physiology of a microgravity environment selected contribution: Effects of spaceflight during pregnancy on labor and birth at 1 G. J. Appl. Physiol..

[41] Burden H.W., Zary J., Alberts J.R. (1999). Effects of space flight on the immunohistochemical demonstration of connexin 26 and connexin 43 in the postpartum uterus of rats. J. Reprod. Fertil..

[42] Oyama J., Platt W.T. (1967). Reproduction and Growth of Mice and Rats under Conditions of Simulated Increased Gravity. Am. J. Physiol..

[43] Plaut K., Maple R.L., Wade C.E., Baer L.A., Ronca A.E. (2003). Effects of hypergravity on mammary metabolic function: Gravity acts as a continuum. J. Appl. Physiol..

[44] Ronca A.E., Baer L.A., Daunton N.G., Wade C.E. (2001). Maternal reproductive experience enhances early postnatal outcome following gestation and birth of rats in hypergravity. Biol. Reprod..

[45] Wu Q.J., Li Y.Y., Tu C., Zhu J., Qian K.Q., Feng T.B., Li C., Wu L., Ma X.X. (2015). Parity and endometrial cancer risk: A meta-analysis of epidemiological studies. Sci. Rep..

[46] Lambe M., Hsieh C.C., Chan H.W., Ekbom A., Trichopoulos D., Adami H.O. (1996). Parity, age at first and last birth, and risk of breast cancer: A population-based study in Sweden. Breast Cancer Res. Treat..

[47] Sung H.K., Ma S.H., Choi J.Y., Hwang Y., Ahn C., Kim B.G., Kim Y.M., Kim J.W., Kang S., Kim J. (2016). The Effect of Breastfeeding Duration and Parity on the Risk of Epithelial Ovarian Cancer: A Systematic Review and Meta-analysis. J. Prev. Med. Public Health.

[48] Jain V., Wotring V.E. (2016). Medically induced amenorrhea in female astronauts. NPJ Microgravity.

[49] Prasad K.N. (1995). Handbook of Radiobiology.

[50] Ogilvy-Stuart A.L., Shalet S.M. (1993). Effect of radiation on the human reproductive system. Environ. Health Perspect..

[51] Marci R., Mallozzi M., Di Benedetto L., Schimberni M., Mossa S., Soave I., Palomba S., Caserta D. (2018). Radiations and female fertility. Reprod. Biol. Endocrinol..

[52] Hanahan D., Weinberg R.A. (2011). Hallmarks of cancer: The next generation. Cell.

[53] Grimm D., Bauer J., Wise P., Krüger M., Simonsen U., Wehland M., Infanger M., Corydon T.J. (2020). The role of SOX family members in solid tumours and metastasis. Semin. Cancer Biol..

[54] Fouad Y.A., Aanei C. (2017). Revisiting the hallmarks of cancer. Am. J. Cancer Res..

[55] Moreno-Villanueva M., Wu H. (2019). Radiation and microgravity—Associated stress factors and carcinogensis. REACH.

[56] Nassef M.Z., Kopp S., Wehland M., Melnik D., Sahana J., Krüger M., Corydon T.J., Oltmann H., Schmitz B., Schütte A. (2019). Real Microgravity Influences the Cytoskeleton and Focal Adhesions in Human Breast Cancer Cells. Int. J. Mol. Sci..

[57] Kopp S., Slumstrup L., Corydon T.J., Sahana J., Aleshcheva G., Islam T., Magnusson N.E., Wehland M., Bauer J., Infanger M. (2016). Identifications of novel mechanisms in breast cancer cells involving duct-like multicellular spheroid formation after exposure to the Random Positioning Machine. Sci. Rep..

[58] Nassef M.Z., Kopp S., Melnik D., Corydon T.J., Sahana J., Krüger M., Wehland M., Bauer T.J., Liemersdorf C., Hemmersbach R. (2019). Short-Term Microgravity Influences Cell Adhesion in Human Breast Cancer Cells. Int. J. Mol. Sci..

[59] Deng B., Liu R., Tian X., Han Z., Chen J. (2019). Simulated microgravity inhibits the viability and migration of glioma via FAK/RhoA/Rock and FAK/Nek2 signaling. In Vitro Cell. Dev. Biol. Anim..

[60] Dietz C., Infanger M., Romswinkel A., Strube F., Kraus A. (2019). Apoptosis Induction and Alteration of Cell Adherence in Human Lung Cancer Cells under Simulated Microgravity. Int. J. Mol. Sci..

[61] Przystupski D., Górska A., Michel O., Podwin A., Śniadek P., Łapczyński R., Saczko J., Kulbacka J. (2021). Testing Lab-on-a-Chip Technology for Culturing Human Melanoma Cells under Simulated Microgravity. Cancers.

[62] Warnke E., Pietsch J., Wehland M., Bauer J., Infanger M., Görög M., Hemmersbach R., Braun M., Ma X., Sahana J. (2014). Spheroid formation of human thyroid cancer cells under simulated microgravity: A possible role of CTGF and CAV1. Cell Commun. Signal..

[63] Riwaldt S., Bauer J., Pietsch J., Braun M., Segerer J., Schwarzwälder A., Corydon T.J., Infanger M., Grimm D. (2015). The Importance of Caveolin-1 as Key-Regulator of Three-Dimensional Growth in Thyroid Cancer Cells Cultured under Real and Simulated Microgravity Conditions. Int. J. Mol. Sci..

[64] Cao Q., Liu W., Wang J., Cao J., Yang H. (2018). A single low dose of Fe ions can cause long-term biological responses in NL20 human bronchial epithelial cells. Radiat. Environ. Biophys..

[65] Kennedy E.M., Powell D.R., Li Z., Bell J.S.K., Barwick B.G., Feng H., McCrary M.R., Dwivedi B., Kowalski J., Dynan W.S. (2018). Galactic Cosmic Radiation Induces Persistent Epigenome Alterations Relevant to Human Lung Cancer. Sci. Rep..

[66] Patel R., Zhang L., Desai A., Hoenerhoff M.J., Kennedy L.H., Radivoyevitch T., La Tessa C., Gerson S.L., Welford S.M. (2020). Protons and High-Linear Energy Transfer Radiation Induce Genetically Similar Lymphomas with High Penetrance in a Mouse Model of the Aging Human Hematopoietic System. Int. J. Radiat. Oncol. Biol. Phys..

[67] Wage J., Ma L., Peluso M., Lamont C., Evens A.M., Hahnfeldt P., Hlatky L., Beheshti A. (2015). Proton irradiation impacts age-driven modulations of cancer progression influenced by immune system transcriptome modifications from splenic tissue. J. Radiat. Res..

[68] Cheema A.K., Suman S., Kaur P., Singh R., Fornace A.J., Datta K. (2014). Long-term differential changes in mouse intestinal metabolomics after γ and heavy ion radiation exposure. PLoS ONE.

[69] Weil M.M., Bedford J.S., Bielefeldt-Ohmann H., Ray F.A., Genik P.C., Ehrhart E.J., Fallgren C.M., Hailu F., Battaglia C.L., Charles B. (2009). Incidence of acute myeloid leukemia and hepatocellular carcinoma in mice irradiated with 1 GeV/nucleon (56)Fe ions. Radiat. Res..

[70] Turker M.S., Grygoryev D., Lasarev M., Ohlrich A., Rwatambuga F.A., Johnson S., Dan C., Eckelmann B., Hryciw G., Mao J.H. (2017). Simulated space radiation-induced mutants in the mouse kidney display widespread genomic change. PLoS ONE.

[71] Ohnishi T., Ohnishi K., Takahashi A., Taniguchi Y., Sato M., Nakano T., Nagaoka S. (2002). Detection of DNA damage induced by space radiation in Mir and space shuttle. J. Radiat. Res..

[72] Lu T., Zhang Y., Wong M., Feiveson A., Gaza R., Stoffle N., Wang H., Wilson B., Rohde L., Stodieck L. (2017). Detection of DNA damage by space radiation in human fibroblasts flown on the International Space Station. Life Sci. Space Res..

[73] Zhang Y., Lu T., Wong M., Wang X., Stodieck L., Karouia F., Story M., Wu H. (2016). Transient gene and microRNA expression profile changes of confluent human fibroblast cells in spaceflight. FASEB J..

[74] Blakely E.A., Kronenberg A. (1998). Heavy-ion radiobiology: New approaches to delineate mechanisms underlying enhanced biological effectiveness. Radiat. Res..

[75] Koontz B.F., Verhaegen F., De Ruysscher D. (2017). Tumour and normal tissue radiobiology in mouse models: How close are mice to mini-humans?. Br. J. Radiol..

[76] Barcellos-Hoff M.H., Blakely E.A., Burma S., Fornace A.J., Gerson S., Hlatky L., Kirsch D.G., Luderer U., Shay J., Wang Y. (2015). Concepts and challenges in cancer risk prediction for the space radiation environment. Life Sci. Space Res..

[77] Cucinotta F.A., To K., Cacao E. (2017). Predictions of space radiation fatality risk for exploration missions. Life Sci. Space Res..

[78] Shuryak I., Fornace A.J., Datta K., Suman S., Kumar S., Sachs R.K., Brenner D.J. (2017). Scaling Human Cancer Risks from Low LET to High LET when Dose-Effect Relationships are Complex. Radiat. Res..

[79] Mishra K.P. (2017). Carcinogenic risk from low-dose radiation exposure is overestimated. J. Radiat. Cancer Res..

[80] Prasad N.R. (2017). Radiation carcinogenesis: Mechanisms and experimental models—A meeting report. J. Radiat. Cancer Res..

[81] Moreno-Villanueva M., Wong M., Lu T., Zhang Y., Wu H. (2017). Interplay of space radiation and microgravity in DNA damage and DNA damage response. NPJ Microgravity.

[82] Shi F., Wang Y.C., Zhao T.Z., Zhang S., Du T.Y., Yang C.B., Li Y.H., Sun X.Q. (2012). Effects of simulated microgravity on human umbilical vein endothelial cell angiogenesis and role of the PI3K-Akt-eNOS signal pathway. PLoS ONE.

[83] Dai Z., Guo F., Wu F., Xu H., Yang C., Li J., Liang P., Zhang H., Qu L., Tan Y. (2014). Integrin αvβ3 mediates the synergetic regulation of core-binding factor α1 transcriptional activity by gravity and insulin-like growth factor-1 through phosphoinositide 3-kinase signaling. Bone.

[84] Najrana T., Sanchez-Esteban J. (2016). Mechanotransduction as an Adaptation to Gravity. Front. Pediatr..

[85] Engelman J.A., Luo J., Cantley L.C. (2006). The evolution of phosphatidylinositol 3-kinases as regulators of growth and metabolism. Nat. Rev. Genet..

[86] Arun R.P., Sivanesan D., Vidyasekar P., Verma R.S. (2017). PTEN/FOXO3/AKT pathway regulates cell death and mediates morphogenetic differentiation of Colorectal Cancer Cells under Simulated Microgravity. Sci. Rep..

[87] Monti N., Masiello M.G., Proietti S., Catizone A., Ricci G., Harrath A.H., Alwasel S.H., Cucina A., Bizzarri M. (2021). Survival Pathways Are Differently Affected by Microgravity in Normal and Cancerous Breast Cells. Int. J. Mol. Sci..

[88] Hybel T.E., Dietrichs D., Sahana J., Corydon T.J., Nassef M.Z., Wehland M., Krüger M., Magnusson N.E., Bauer J., Utpatel K. (2020). Simulated Microgravity Influences VEGF, MAPK, and PAM Signaling in Prostate Cancer Cells. Int. J. Mol. Sci..

[89] Sarkar R., Pampaloni F. (2022). In Vitro Models of Bone Marrow Remodelling and Immune Dysfunction in Space: Present State and Future Directions. Biomedicines.

[90] Elgart S.R., Little M.P., Chappell L.J., Milder C.M., Shavers M.R., Huff J.L., Patel Z.S. (2018). Radiation Exposure and Mortality from Cardiovascular Disease and Cancer in Early NASA Astronauts. Sci. Rep..

[91] Di Trolio R., Di Lorenzo G., Fumo B., Ascierto P.A. (2015). Cosmic radiation and cancer: Is there a link?. Future Oncol..

[92] Rahimian N., Razavi Z.S., Aslanbeigi F., Mirkhabbaz A.M., Piroozmand H., Shahrzad M.K., Hamblin M.R., Mirzaei H. (2021). Non-Coding RNAs Related to Angiogenesis in Gynecological Cancer. Gynecol. Oncol..

[93] Di Fiore R., Suleiman S., Pentimalli F., O’Toole S.A., O’Leary J.J., Ward M.P., Conlon N.T., Sabol M., Ozretić P., Erson-Bensan A.E. (2021). Could MicroRNAs Be Useful Tools to Improve the Diagnosis and Treatment of Rare Gynecological Cancers? A Brief Overview. Int. J. Mol. Sci..

[94] Mandilaras V., Karakasis K., Clarke B., Amit Oza A., Lheureux S. (2017). Rare tumors in gynaecological cancers and the lack of therapeutic options and clinical trials. Expert Opin. Orphan Drugs.

[95] Xie W., Sun H., Li X., Lin F., Wang Z., Wang X. (2021). Ovarian cancer: Epigenetics, drug resistance, and progression. Cancer Cell Int..

[96] Hosseini E.S., Meryet-Figuiere M., Sabzalipoor H., Kashani H.H., Nikzad H., Asemi Z. (2017). Dysregulated expression of long noncoding RNAs in gynecologic cancers. Mol. Cancer.

[97] Mota A., Oltra S.S., Moreno-Bueno G. (2020). Insight updating of the molecular hallmarks in ovarian carcinoma. EJC Suppl..

[98] Vaughan S., Coward J.I., Bast R.C., Berchuck A., Berek J.S., Brenton J.D., Coukos G., Crum C.C., Drapkin R., Etemadmoghadam D. (2011). Rethinking ovarian cancer: Recommendations for improving outcomes. Nat. Rev. Cancer.

[99] Bowtell D.D., Böhm S., Ahmed A.A., Aspuria P.J., Bast R.C., Beral V., Berek J.S., Birrer M.J., Blagden S., Bookman M.A. (2015). Rethinking ovarian cancer II: Reducing mortality from high-grade serous ovarian cancer. Nat. Rev. Cancer.

[100] Siegel R.L., Miller K.D., Jemal A. (2019). Cancer statistics, 2019. CA Cancer J. Clin..

[101] Siegel R.L., Miller K.D., Fuchs H.E., Jemal A. (2021). Cancer Statistics, 2021. CA Cancer J. Clin..

[102] Hull R., Mbele M., Makhafola T., Hicks C., Wang S.M., Reis R.M., Mehrotra R., Mkhize-Kwitshana Z., Kibiki G., Bates D.O. (2020). Cervical cancer in low and middle-income countries. Oncol. Lett..

[103] Frazer I.H. (2004). Prevention of cervical cancer through papillomavirus vaccination. Nat. Rev. Immunol..

[104] Kokka F., Bryant A., Brockbank E., Jeyarajah A. (2014). Surgical treatment of stage IA2 cervical cancer. Cochrane Database Syst. Rev..

[105] Louie K.S., de Sanjosé S., Diaz M., Castellsagué X., Herrero R., Meijer C.J., Shah K., Franceschi S., Muñoz N., Bosch F.X. (2009). International Agency for Research on Cancer Multicenter Cervical Cancer Study Group. Early age at first sexual intercourse and early pregnancy are risk factors for cervical cancer in developing countries. Br. J. Cancer.

[106] Liu Z.C., Liu W.D., Liu Y.H., Ye X.H., Chen S.D. (2015). Multiple Sexual Partners as a Potential Independent Risk Factor for Cervical Cancer: A Meta-analysis of Epidemiological Studies. Asian Pac. J. Cancer Prev..

[107] Husain R.S., Ramakrishnan V. (2015). Global Variation of Human Papillomavirus Genotypes and Selected Genes Involved in Cervical Malignancies. Ann. Glob. Health.

[108] Yi Y., Fang Y., Wu K., Liu Y., Zhang W. (2020). Comprehensive gene and pathway analysis of cervical cancer progression. Oncol. Lett..

[109] Roura E., Castellsagué X., Pawlita M., Travier N., Waterboer T., Margall N., Bosch F.X., de Sanjosé S., Dillner J., Gram I.T. (2014). Smoking as a major risk factor for cervical cancer and pre-cancer: Results from the EPIC cohort. Int. J. Cancer.

[110] Peirson L., Fitzpatrick-Lewis D., Ciliska D., Warren R. (2013). Screening for cervical cancer: A systematic review and meta-analysis. Syst. Rev..

[111] Xu F., Li Y., Fan L., Ma J., Yu L., Yi H., Chen X., Wei W., Wu P., Liang L. (2018). Preoperative SCC-Ag and thrombocytosis as predictive markers for pelvic lymphatic metastasis of squamous cervical cancer in early FIGO stage. J. Cancer.

[112] Nanthamongkolkul K., Hanprasertpong J. (2018). Predictive Factors of Pelvic Lymph Node Metastasis in Early-Stage Cervical Cancer. Oncol. Res. Treat..

[113] Wright J.D., Huang Y., Ananth C.V., Tergas A.I., Duffy C., Deutsch I., Burke W.M., Hou J.Y., Neugut A.I., Hershman D.L. (2015). Influence of treatment center and hospital volume on survival for locally advanced cervical cancer. Gynecol. Oncol..

[114] Vaccarella S., Lortet-Tieulent J., Plummer M., Franceschi S., Bray F. (2013). Worldwide trends in cervical cancer incidence: Impact of screening against changes in disease risk factors. Eur. J. Cancer.

[115] Jemal A., Bray F., Center M.M., Ferlay J., Ward E., Forman D. (2011). Global cancer statistics. CA Cancer J. Clin..

[116] Ferlay J., Soerjomataram I., Dikshit R., Eser S., Mathers C., Rebelo M., Parkin D.M., Forman D., Bray F. (2015). Cancer incidence and mortality worldwide: Sources, methods and major patterns in GLOBOCAN 2012. Int. J. Cancer.

[117] Di Fiore R., Suleiman S., Drago-Ferrante R., Felix A., O’Toole S.A., O’Leary J.J., Ward M.P., Beirne J., Yordanov A., Vasileva-Slaveva M. (2021). LncRNA *MORT* (*ZNF667-AS1*) in Cancer—Is There a Possible Role in Gynecological Malignancies?. Int. J. Mol. Sci..

[118] Yasin H.K., Taylor A.H., Ayakannu T. (2021). A Narrative Review of the Role of Diet and Lifestyle Factors in the Development and Prevention of Endometrial Cancer. Cancers.

[119] Lu K.H., Broaddus R.R. (2020). Endometrial Cancer. N. Engl. J. Med..

[120] Evrard C., Alexandre J. (2021). Predictive and Prognostic Value of Microsatellite Instability in Gynecologic Cancer (Endometrial and Ovarian). Cancers.

[121] Lax S.F. (2004). Molecular genetic pathways in various types of endometrial carcinoma: From a phenotypical to a molecular-based classification. Virchows Arch..

[122] Lax S.F., Kendall B., Tashiro H., Slebos R.J., Hedrick L. (2000). The frequency of p53, K-ras mutations, and microsatellite instability differs in uterine endometrioid and serous carcinoma: Evidence of distinct molecular genetic pathways. Cancer.

[123] Morice P., Leary A., Creutzberg C., Abu-Rustum N., Darai E. (2016). Endometrial cancer. Lancet.

[124] Di Tucci C., Capone C., Galati G., Iacobelli V., Schiavi M.C., Di Donato V., Muzii L., Panici P.B. (2019). Immunotherapy in endometrial cancer: New scenarios on the horizon. J. Gynecol. Oncol..

[125] Siegel R.L., Miller K.D., Jemal A. (2020). Cancer statistics, 2020. CA Cancer J. Clin..

[126] Daling J.R., Madeleine M.M., Schwartz S.M., Shera K.A., Carter J.J., McKnight B., Porter P.L., Galloway D.A., McDougall J.K., Tamimi H. (2002). A population-based study of squamous cell vaginal cancer: HPV and cofactors. Gynecol. Oncol..

[127] Saraiya M., Watson M., Wu X., King J.B., Chen V.W., Smith J.S., Giuliano A.R. (2008). Incidence of in situ and invasive vulvar cancer in the US, 1998–2003. Cancer.

[128] Shah C.A., Goff B.A., Lowe K., Peters W.A., Li C.I. (2009). Factors affecting risk of mortality in women with vaginal cancer. Obstet. Gynecol..

[129] How J.A., Jazaeri A.A., Soliman P.T., Fleming N.D., Gong J., Piha-Paul S.A., Janku F., Stephen B., Naing A. (2021). Pembrolizumab in vaginal and vulvar squamous cell carcinoma: A case series from a phase II basket trial. Sci. Rep..

[130] Krüger M., Bauer J., Grimm D., Ruyters G., Betzel C., Grimm D. (2017). Cancer Research in Space. Biotechnology in Space.

[131] Grimm D., Schulz H., Krüger M., Cortés-Sánchez J.L., Egli M., Kraus A., Sahana J., Corydon T.J., Hemmersbach R., Wise P.M. (2022). The Fight against Cancer by Microgravity: The Multicellular Spheroid as a Metastasis Model. Int. J. Mol. Sci..

[132] Becker J.L., Souza G.R. (2013). Using space-based investigations to inform cancer research on Earth. Nat. Rev. Cancer.

[133] Mehner C., Krishnan S., Chou J., Freeman M.L., Freeman W.D., Patel T., Turnbull M.T. (2021). Real versus simulated galactic cosmic radiation for investigating cancer risk in the hematopoietic system-are we comparing apples to apples?. Life Sci. Space Res..

[134] Becker J.L., Prewett T.L., Spaulding G.F., Goodwin T.J. (1993). Three-dimensional growth and differentiation of ovarian tumor cell line in high aspect rotating-wall vessel: Morphologic and embryologic considerations. J. Cell Biochem..

[135] Goodwin T.J., Prewett T.L., Spaulding G.F., Becker J.L. (1997). Three-dimensional culture of a mixed mullerian tumor of the ovary: Expression of in vivo characteristics. In Vitro Cell. Dev. Biol. Anim..

[136] Hammond D.K., Becker J., Elliott T.F., Holubee K., Baker T.L., Love J.E. (2005). Antigenic protein in microgravity-grown human mixed Mullerian ovarian tumor (LN1) cells preserved in RNA stabilizing agent. Gravit. Space Biol. Bull..

[137] Przystupski D., Górska A., Szewczyk A., Drag-Zalensinka M., Kulbacka J. (2021). 3D Clinorotation Affects Drug Sensitivity of Human Ovarian Cancer Cells. Microgravity Sci. Technol..

[138] Mukhopadhyay S., Saha R., Palanisamy A., Ghosh M., Biswas A., Roy S., Pal A., Sarkar K., Bagh S. (2016). A systems biology pipeline identifies new immune and disease related molecular signatures and networks in human cells during microgravity exposure. Sci. Rep..

[139] Watanabe H., Ogiu T., Nishizaki M., Fujimoto N., Kido S., Ishimura Y., Shiraki K., Kuramoto K., Hirata S., Shoji S. (1998). Induction of ovarian tumors by heavy ion irradiation in B6C3F1 mice. Oncol. Rep..

[140] Watanabe H., Ogiu T., Nishimura M., Masaoka Y., Kurosumi M., Takahashi T., Oguri T., Shoji S., Katoh O. (1998). Comparison of tumorigenesis between accelerated heavy ion and X-ray in B6C3F1 mice. J. Radiat. Res..

[141] Nitta Y., Hoshi M. (2003). Relationship between oocyte apoptosis and ovarian tumours induced by high and low LET radiations in mice. Int. J. Radiat. Biol..

[142] Mishra B., Lawson G.W., Ripperdan R., Ortiz L., Luderer U. (2018). Charged-Iron-Particles Found in Galactic Cosmic Rays are Potent Inducers of Epithelial Ovarian Tumors. Radiat. Res..

[143] Chopra V., Dinh T.V., Hannigan E.V. (1997). Three-dimensional endothelial-tumor epithelial cell interactions in human cervical cancers. In Vitro Cell. Dev. Biol. Anim..

[144] Kelly S.E., Di Benedetto A., Greco A., Howard C.M., Sollars V.E., Primerano D.A., Valluri J.V., Claudio P.P. (2010). Rapid selection and proliferation of CD133+ cells from cancer cell lines: Chemotherapeutic implications. PLoS ONE.

[145] Zhang Z.J., Tong Y.Q., Wang J.J., Yang C., Zhou G.H., Li Y.H., Xie P.L., Hu J.Y., Li G.C. (2011). Spaceflight alters the gene expression profile of cervical cancer cells. Chin. J. Cancer.

[146] Guo F., Li Y., Liu Y., Huang J., Zhang Z., Wang J., Li Y., Hu J., Li G. (2012). Identification of genes associated with tumor development in CaSki cells in the cosmic space. Mol. Biol. Rep..

[147] de Martel C., Plummer M., Vignat J., Franceschi S. (2017). Worldwide burden of cancer attributable to HPV by site, country and HPV type. Int. J. Cancer.

[148] Maglennon G.A., Doorbar J. (2012). The biology of papillomavirus latency. Open Virol. J..

[149] Guidry J.T., Scott R.S. (2017). The interaction between human papillomavirus and other viruses. Virus Res..

[150] Mehta S.K., Laudenslager M.L., Stowe R.P., Crucian B.E., Feiveson A.H., Sams C.F., Pierson D.L. (2017). Latent virus reactivation in astronauts on the international space station. NPJ Microgravity.

[151] Bigley A.B., Agha N.H., Baker F.L., Spielmann G., Kunz H.E., Mylabathula P.L., Rooney B.V., Laughlin M.S., Mehta S.K., Pierson D.L. (2019). NK cell function is impaired during long-duration spaceflight. J. Appl. Physiol..

[152] Martinez E.M., Yoshida M.C., Candelario T.L., Hughes-Fulford M. (2015). Spaceflight and simulated microgravity cause a significant reduction of key gene expression in early T-cell activation. Am. J. Physiol. Regul. Integr. Comp. Physiol..

[153] Rooney B.V., Crucian B.E., Pierson D.L., Laudenslager M.L., Mehta S.K. (2019). Herpes Virus Reactivation in Astronauts during Spaceflight and Its Application on Earth. Front. Microbiol..

[154] Jennings R.T., Baker E.S. (2000). Gynecological and reproductive issues for women in space: A review. Obstet. Gynecol. Surv..

[155] Cho H.J., Baek M.O., Khaliq S.A., Chon S.J., Son K.H., Lee S.H., Yoon M.S. (2019). Microgravity inhibits decidualization via decreasing Akt activity and FOXO3a expression in human endometrial stromal cells. Sci. Rep..

[156] Grun B., Benjamin E., Sinclair J., Timms J.F., Jacobs I.J., Gayther S.A., Dafou D. (2009). Three-dimensional in vitro cell biology models of ovarian and endometrial cancer. Cell Prolif..

[157] Palumbo G., Varriale L., Paba V., Sasso A., Crescenzi E., Gialanella G., Grossi G., Pugliese M.G., Scampoli P. (2001). Effect of space radiation on expression of apoptosis-related genes in endometrial cells: A preliminary study. Phys. Med..

[158] Jennings R., Baker E., Barratt M.R., Pool S.L. (2008). Gynecologic and Reproductive Concerns. Principles of Clinical Medicine for Space Flight.

[159] Werneth C.M., Slaba T.C., Huff J.L., Patel Z.S., Simonsen L.C. (2022). Medical Countermeasure Requirements to Meet NASA’s Space Radiation Permissible Exposure Limits for a Mars Mission Scenario. Health Phys..

[160] Kim M.J., Kim J.J., Kim S.M. (2016). Endometrial evaluation with transvaginal ultrasonography for the screening of endometrial hyperplasia or cancer in premenopausal and perimenopausal women. Obstet. Gynecol. Sci..

[161] Steller J.G., Blue R., Zahner C., Frisch E.H., Bayuse T., Auñon-Chancellor S., Jennings R.T. (2021). Menstrual management considerations in the space environment. Reach.

[162] Bogomolov V.V., Castrucci F., Comtois J.M., Damann V., Davis J.R., Duncan J.M., Johnston S.L., Gray G.W., Grigoriev A.I., Koike Y. (2007). International Space Station medical standards and certification for space flight participants. Aviat. Space Environ. Med..

[163] Barthel J., Sarigul-Klijn N. (2019). A review of radiation shielding needs and concepts for space voyages beyond Earth’s magnetic influence. Prog. Aerosp. Sci..

[164] Montesinos C.A., Khalid R., Cristea O., Greenberger J.S., Epperly M.W., Lemon J.A., Boreham D.R., Popov D., Gorthi G., Ramkumar N. (2021). Space Radiation Protection Countermeasures in Microgravity and Planetary Exploration. Life.

[165] Reliene R., Pollard J.M., Sobol Z., Trouiller B., Gatti R.A., Schiestl R.H. (2009). N-acetyl cysteine protects against ionizing radiation-induced DNA damage but not against cell killing in yeast and mammals. Mutat. Res..

[166] Wang Y., Li L., Fan L.H., Jing Y., Li J., Ouyang Y.C., Wang Z.B., Hou Y., Sun Q.Y. (2019). N-acetyl-L-cysteine (NAC) delays post-ovulatory oocyte aging in mouse. Aging.

[167] Mendelev N., Mehta S.L., Idris H., Kumari S., Li P.A. (2012). Selenite stimulates mitochondrial biogenesis signaling and enhances mitochondrial functional performance in murine hippocampal neuronal cells. PLoS ONE.

[168] McKenzie R.C., Beckett G.J., Arthur J.R., Hatfield D.L., Berry M.J., Gladyshev V.N. (2006). Effects of selenium on immunity and aging. Selenium: Its Molecular Biology and Role in Human Health.

[169] Suzuki T., Suzuki T., Wada T., Saigo K., Watanabe K. (2002). Taurine as a constituent of mitochondrial tRNAs: New insights into the functions of taurine and human mitochondrial diseases. EMBO J..

[170] Pisoschi A.M., Pop A. (2015). The role of antioxidants in the chemistry of oxidative stress: A review. Eur. J. Med. Chem..

[171] Hashimoto T., Horikawa D.D., Saito Y., Kuwahara H., Kozuka-Hata H., Shin-I T., Minakuchi Y., Ohishi K., Motoyama A., Aizu T. (2016). Extremotolerant tardigrade genome and improved radiotolerance of human cultured cells by tardigrade-unique protein. Nat. Commun..

[172] Sanzari J.K., Krigsfeld G.S., Shuman A.L., Diener A.K., Lin L., Mai W., Kennedy A.R. (2015). Effects of a granulocyte colony stimulating factor, Neulasta, in mini pigs exposed to total body proton irradiation. Life Sci. Space Res..

[173] Bahamondes L., Valeria Bahamondes M., Shulman L.P. (2015). Non-contraceptive benefits of hormonal and intrauterine reversible contraceptive methods. Hum. Reprod. Update.

[174] Asthana S., Busa V., Labani S. (2020). Oral contraceptives use and risk of cervical cancer—A systematic review & meta-analysis. Eur. J. Obstet. Gynecol. Reprod. Biol..

[175] Iversen L., Fielding S., Lidegaard Ø., Hannaford P.C. (2021). Contemporary hormonal contraception and cervical cancer in women of reproductive age. Int. J. Cancer.

[176] Cogliano V., Baan R., Straif K., Grosse Y., Secretan B., El Ghissassi F., WHO International Agency for Research on Cancer (2005). Carcinogenicity of human papillomaviruses. Lancet Oncol..

[177] Cibula D., Gompel A., Mueck A.O., La Vecchia C., Hannaford P.C., Skouby S.O., Zikan M., Dusek L. (2010). Hormonal contraception and risk of cancer. Hum. Reprod. Update.

[178] Mørch L.S., Hannaford P.C., Lidegaard Ø. (2018). Contemporary Hormonal Contraception and the Risk of Breast Cancer. N. Engl J. Med..

[179] Conz L., Mota B.S., Bahamondes L., Teixeira Dória M., Françoise Mauricette Derchain S., Rieira R., Sarian L.O. (2020). Levonorgestrel-releasing intrauterine system and breast cancer risk: A systematic review and meta-analysis. Acta Obstet. Gynecol. Scand..

[180] Greenberger J.S. (2009). Radioprotection. In Vivo.

[181] Belli M., Tabocchini M.A. (2020). Ionizing Radiation-Induced Epigenetic Modifications and Their Relevance to Radiation Protection. Int. J. Mol. Sci..

[182] Daly M.B., Pilarski R., Yurgelun M.B., Berry M.P., Buys S.S., Dickson P., Domchek S.M., Elkhanany A., Friedman S., Garber J.E. (2020). NCCN Guidelines Insights: Genetic/Familial High-Risk Assessment: Breast, Ovarian, and Pancreatic, Version 1.2020. J. Natl. Compr. Canc. Netw..

[183] Nuñez J.K., Chen J., Pommier G.C., Cogan J.Z., Replogle J.M., Adriaens C., Ramadoss G.N., Shi Q., Hung K.L., Samelson A.J. (2021). Genome-wide programmable transcriptional memory by CRISPR-based epigenome editing. Cell.

[184] Liu G., Lin Q., Jin S., Gao C. (2022). The CRISPR-Cas toolbox and gene editing technologies. Mol. Cell..

[185] Berrios D.C., Galazka J., Grigorev K., Gebre S., Costes S.V. (2021). NASA GeneLab: Interfaces for the exploration of space omics data. Nucleic Acids Res..

[186] Datta K., Hyduke D.R., Suman S., Moon B.H., Johnson M.D., Fornace A.J. (2012). Exposure to ionizing radiation induced persistent gene expression changes in mousemammary gland. Radiat. Oncol..

